# NuConf: a rotamer library for DNA and RNA and its implementation in the protein design software MUMBO

**DOI:** 10.1038/s41598-026-52380-3

**Published:** 2026-05-26

**Authors:** Marharyta O. Makarova, Martin T. Stiebritz, Derman Basturk, Birthe Lemke, Beatrix Süss, Yves A. Muller

**Affiliations:** 1 Division of Biotechnology, Department of Biology, Friedrich-Alexander-Universität Erlangen-Nürnberg (FAU), 91052 Erlangen, Germany; 2 Max Planck Center for Physics and Medicine, 91054 Erlangen, Germany; 3 Department of Biology, Technische Universität Darmstadt, 64287 Darmstadt, Germany; 4 Centre for Synthetic Biology, Technische Universität Darmstadt, 64287 Darmstadt, Germany

**Keywords:** Biochemistry, Chemistry, Computational biology and bioinformatics, Structural biology

## Abstract

Current AI tools for designing macromolecules have been struggling with DNA and RNA structure prediction and design as significantly less experimental training data are available for nucleic acids than for proteins. Therefore, developing alternative approaches remains of interest. MUMBO, a program for designing protein–protein interactions and protein–ligand-binding pockets, uses side-chain-packing algorithms to select from alternative amino acids and conformers generated on a fixed backbone with the help of rotamer libraries. MUMBO identifies the most favourable combinations based on the lowest overall energy. In order to extend the program’s capabilities to designing nucleic acids, we developed NuConf, a discrete pseudorotational angle-dependent nucleoside-specific rotamer library. We derived NuConf by statistically analysing pseudorotational and dihedral angles of more than 175,000 nucleotides from experimental structures and validated it by rebuilding more than 20,000 nucleotides in a custom dataset. Strikingly, our approach predicts nucleotides at least as accurately as amino acids. We show that the implementation of the NuConf library in MUMBO enables modelling and designing DNA and RNA sequences on a fixed backbone together with protein-nucleic acid interaction interfaces. Because its approach and format are program agnostic, NuConf can be used by other molecular design frameworks as well.

**Supplementary Information:**

The online version contains supplementary material available at 10.1038/s41598-026-52380-3.

## Introduction

The computational design of biological macromolecules with novel functions is a topical subject in protein science. With the advent of AI-based technologies such as generative diffusion techniques, the field has significantly progressed in recent years^1,2^. However, whereas these AI-inferred models appear of significant use for the design of novel proteins and protein–protein interactions, it remains currently unclear whether these models are of sufficient quality for the design of polynucleotides as well as protein-DNA and protein–RNA complexes^3^. A present shortcoming is that the number of experimentally determined polynucleotide or protein-polynucleotide complex structures available for training AI models is one to two orders of magnitude smaller than in the case of proteins^3^. Hence, tools for designing and evaluating structures using all-atom energy interaction calculations remain valuable for the design of novel polynucleotides and protein-polynucleotide interaction interfaces.

Computer programs such as MUMBO make use of side-chain-packing algorithms for the design of new ligand-binding pockets and protein–protein interaction interfaces^4^. They generate first a highly diverse set of amino acids and side-chain orientations, which are then displayed from a rigid protein backbone frame. Subsequently, by means of energy calculations and combinatorial selection, a combination of amino acids and side-chain orientations displaying the lowest overall energy is identified. In order to restrict the combinatorial challenge, side-chain orientations are modelled using discrete individual rotamers with each rotamer representing a single energetically favoured conformation. Each side-chain rotamer is defined by a set of dihedral angles, and the amino acid-specific values of these angles are retrieved from rotamer libraries^5^. To date, various rotamer libraries have been developed for amino acids^6–9^. In the case of nucleotides, research focused on libraries that classified nucleotides based on the conformation of the entire nucleotide, i.e. the phosphate-sugar backbone conformation and nucleobase orientation^10^. Recent research converged on libraries that clustered the preferred conformations of dinucleotides or nucleotide steps instead of single nucleotides^11^. The nucleotide conformer (NtC) library classifies RNA and DNA dinucleotides into 96 + 1 classes^12^. However, it is not entirely clear how these libraries can be incorporated in a unified framework that allows for the equivalent modelling of DNA, RNA, proteins and polynucleotide-protein complexes with side-chain-packing algorithms. Nucleotide libraries suitable for side-chain-packing algorithms remain scarce and, so far, appear to be limited to RNA only^13,14^.

In the present study, we analysed more than 175,000 nucleotides in order to derive a comprehensive nucleoside rotamer library, i.e., defined sets of preferred nucleobase orientations for the nucleotides dA, dT, dG, dC, A, U, G, and C. To do so, we focused on the analysis of (i) the sugar ring puckering as characterised by the pseudorotation angle P and (ii) the dihedral angle χ that defines the orientation of the nucleobase with respect to the sugar ring (Fig. S1)^15,16^. The observation that in nucleotides P angles and χ angles are highly correlated lead us to generate a P angle-dependent rotamer library, termed NuConf. In this library, two separate sets of preferred χ angles and rotamer probabilities are listed for each nucleotide depending on whether the sugar moiety adopts a 2’-endo or 3’-endo-like conformation. The library thus resembles protein backbone conformation-dependent amino acid rotamer libraries widely used in protein design programs^5^. We show that with this rotamer library, nucleotides can be modelled with accuracies even slightly exceeding those achieved for amino acids.

## Results

### P angle distributions—statistical analysis of ribose/deoxyribose conformations in nucleic acids

The pseudorotation angle P maps the entirety of envelope and twist ring puckering conformations of a ribose/deoxyribose ring onto a single angular value^16^. P is calculated from the five endocyclic dihedral angles ν0, ν1, ν2, ν3, and ν4, and all possible combinations of these angles can be assigned uniformly to P (Fig. S1). In addition, the ring puckering is characterized by the magnitude of the puckering as assessed by the pseudorotation amplitude τ_M_^16,17^. τ_M_ describes the upper limit of the endocyclic torsion angles and provides a measure for the extent of the displacement of the sugar atoms from the sugar plane.

**Fig. 1:**
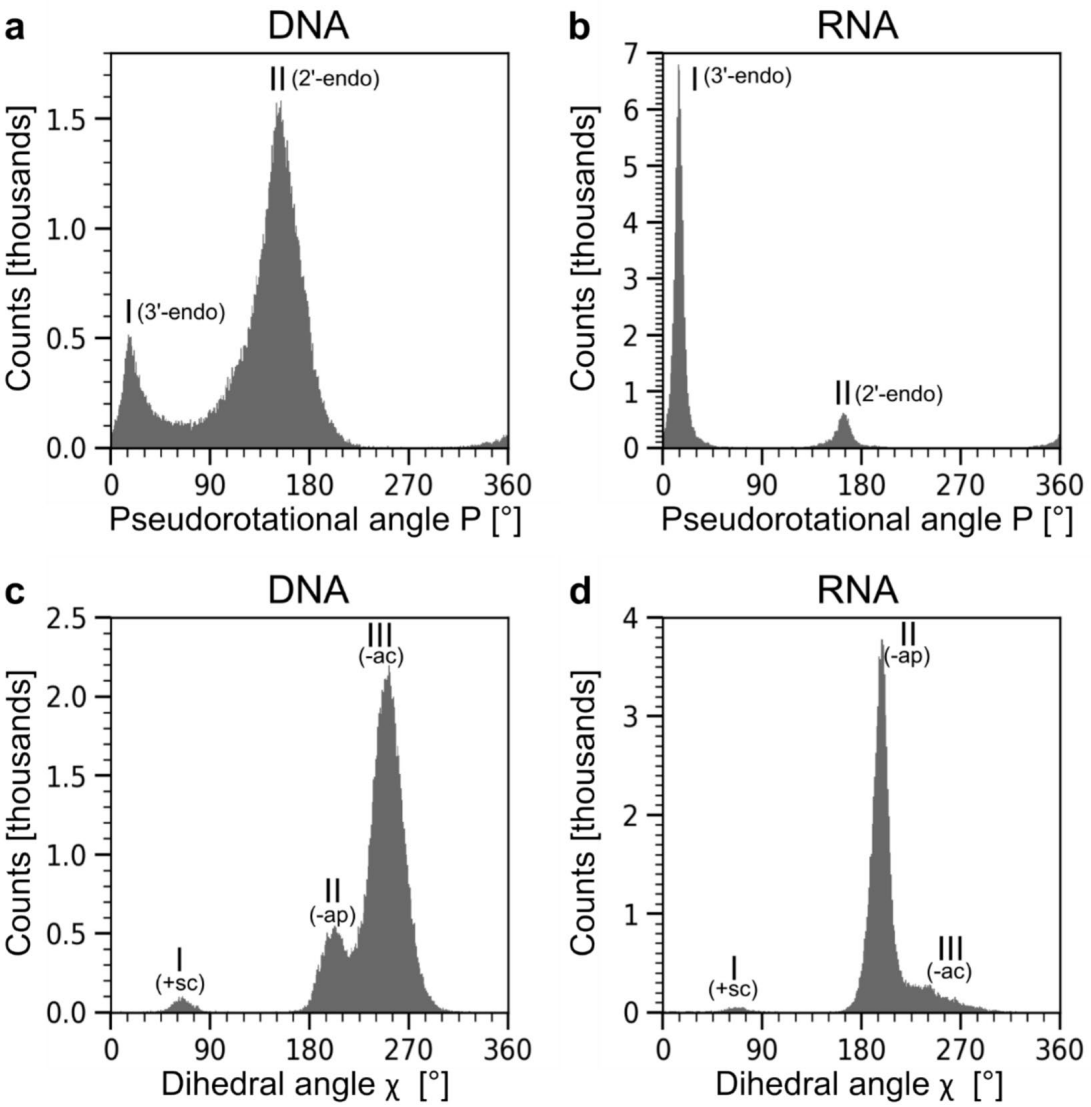
The distribution of pseudorotational angle P and torsion angle χ in DNA and RNA. (**a**) P angle distribution in deoxyribonucleotides and (**b**) in ribonucleotides. (**c**) χ angle distribution in deoxyribonucleotides and (**d**) in ribonucleotides.

The observed P angle distributions look significantly different for deoxyribonucleotides and ribonucleotides (Fig. 1), but two distinct peaks can be observed in each case. In DNA structures, there is no clear separation between peaks I (≈ 30°) and II (≈ 150°) since both peaks overlap considerably at around 80° (Fig. 1a). Peak II is also considerably more populated and broader than peak I. In RNA structures, peaks I (mean value ≈ 15°) and II (≈ 160°) are clearly separated, with peak I being dominant (Fig. 1b). The pseudorotation amplitude τ distributions display maxima at 37° (deoxyribonucleotides) and 40° (ribonucleotides) (Fig. S2).

Peak I of the P angle distributions corresponds to the ^3^E or 3’-endo conformation of the ribose/deoxyribose ring (expected P value of 18°), which is a hallmark of nucleotide conformations in the helical A form of double-stranded polynucleotides, adopted preferentially in double-stranded RNA molecules^15^. Conversely, peak II corresponds to the ^2^E or 2’-endo ribose/deoxyribose conformation (expected P value of 162°), which is a characteristic for the polynucleotide B form, favoured in double-stranded DNA molecules. However, the peak values deviate slightly from expectations, especially for deoxyribonucleotides. Peak II (≈ 150°) appears closer to the ^2^_1_T conformation (2’-endo,1’-exo twist conformation, expected P value = 144°) than to the ^2^E conformation. Overall, the P angle distributions show that the sugar ring in nucleotides adopts a range of conformations in addition to the textbook ^2^E and ^3^E conformations^15^. Conversely, a range of ring puckering conformations will be required to model the nucleotide conformations observed in experimentally determined structures.

Interestingly, when comparing P angle distributions collated separately from data of protein-free polynucleotides and protein-polynucleotide complex structures, it becomes apparent that in case of the latter, the 2’-endo ribose/deoxyribose conformation (peak II) becomes more populated in comparison to the 3’-endo conformation (peak I) (Fig. S3). This shift upon protein binding is especially obvious in DNA–protein complexes.

### χ angle distributions—statistical analysis of nucleobase orientations

The χ angle describes the rotation of the nucleobase around the N-glycosidic bond and its orientation with respect to the sugar moiety^15^. The observed χ angle distributions are multimodal for both deoxyribonucleotides and ribonucleotides and feature three main peaks (Fig. 1c, d). Peak I (≈ 65°) corresponds to the + *synclinal* (+ sc) orientation, commonly referred to as syn-nucleobase orientation in nucleotides (Fig. S1c). Purine moieties famously adopt this orientation in the Z-form of DNA^18^. The syn-orientation of guanines also occurs preferentially in G-rich anti-parallel quadruplexes^19^. Peak II (≈ 200°, -ap) and peak III (≈ 250°, -ac) correspond to nucleobases in -*anti* orientation and are further specified by the terms -anti*periplanar* (-ap) and -anti*clinal* (-ac), respectively^15^.

As the relative peak heights in Fig. 1c, d show, the -ap orientation is clearly preferred in ribonucleotides, whereas the -ac orientation is favoured in deoxyribonucleotides. In analogy to the P angle distributions presented above, we also noted distinct changes in the χ angle distributions going from protein-*free* to protein-*bound* nucleic acids. For DNA-containing protein complexes, the -ac conformation (peak III) becomes significantly more frequent at the expense of the -ap conformation (peak II) (Fig. S3).

### Correlations between P and χ

**Fig. 2:**
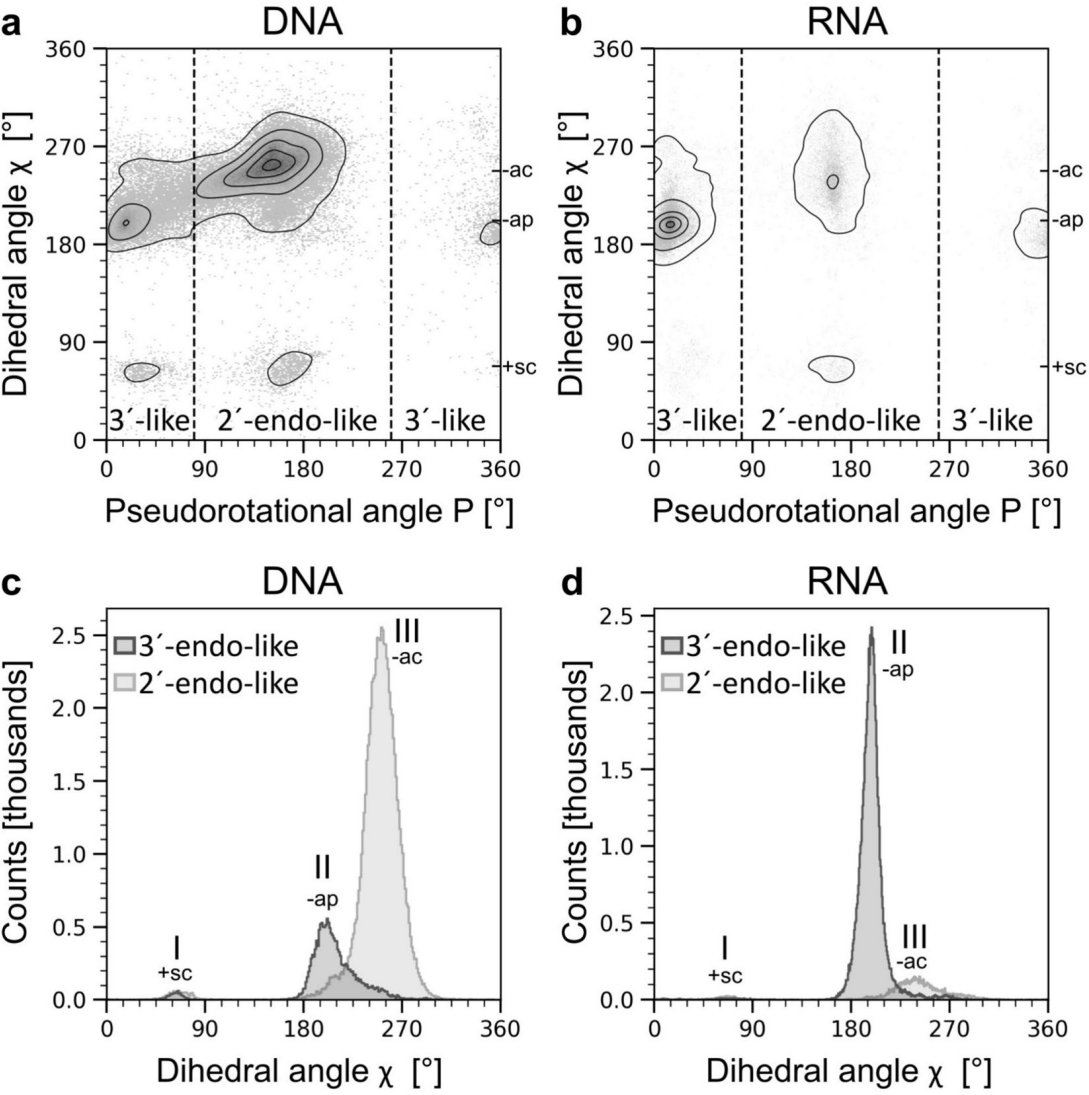
Correlation between pseudorotational angle P and torsion angle χ in nucleotides. (**a**) Scatter plot of P and χ values derived from DNA-containing structures. (**b**) Scatter plot of P and χ values derived from RNA-containing structures. (**c**) P angle-dependent distribution of angle χ in deoxyribonucleotides. (**d**) P angle-dependent distribution of angle χ in ribonucleotides. In (a and b), the density contour lines enclose 95%, 75%, 50%, 25%, and 5% of all data points.

Our datasets reveal a clear correlation between P and χ, which implies that the conformational space available to individual bases in nucleic acids is strongly affected by the conformational states of the associated sugar moieties (circular correlation coefficients of 0.62 and 0.56 for DNA and RNA, respectively^20^). To investigate this interdependency, we generated P-χ plots (Fig. 2a, b).

When describing nucleotides based on their respective P angles as either 2´-endo-like (80° < P < 260°) *or* 3´-endo-like (0° < P < 80° and 260° < P < 360°), it becomes obvious that 2´-endo-like sugar moieties are more strongly associated with bases in the -ac orientation, while the -ap orientation is more frequently represented by 3´-endo-like sugars. These differences in conformational preference are more pronounced for the RNA dataset, which shows two clearly separated clusters (Fig. 2b). The DNA dataset, on the other hand, features considerable overlap between the two clusters (Fig. 2a). The P-χ plot also reveals that for the 2´-endo-related data, the χ angle subset corresponding to nucleobase orientation + sc is more strongly populated than for the 3´-endo-related data (Fig. 2a, b). We subsequently subdivided all nucleotides into either 2’-endo or 3’-endo-like and calculated separate χ angle distributions for each subset (Fig. 2c, d). Upon introduction of this classification step, the distributions become largely uni- or bimodal. The P-χ angle correlation has already been described in the literature before^15^. However, the nucleotide datasets used in the present study are considerably larger, thereby providing improved statistics^21^.

We applied the 2’-endo/3’-endo-specific sorting to each nucleotide type separately and calculated individual, i.e., nucleotide-specific, P-dependent χ distributions (Figs. S4 and S5). The distributions reveal interesting relationships. For example: while the + sc peak is most pronounced for dG nucleotides, it can also be observed for other purine-containing nucleotides, while for pyrimidines, it is almost completely absent. This agrees with the fact that in Z-DNA, which is composed of dGdC-repeats, dG adopts a syn and dC an anti conformation^18^. Furthermore, specifically for dC, there is a considerable amount of overlap in the -ac and -ap region (Fig. S4b). The pronounced differences in the P-dependent χ distributions encouraged us to derive P angle-dependent rotamer libraries for each type of nucleoside.

### Compilation of nucleoside rotamer libraries

With the goal to derive a P angle-dependent rotamer library in a statistically sound manner, we applied the univariate Gaussian mixture model (GMM)^21,22^. In this model, a multimodal distribution is described by a finite linear combination of Gaussian functions (components) whose parameters are optimized to best model the underlying data. The optimal number of Gaussian components can then be assessed by calculating the so-called Bayesian information criterion (BIC)^23^. When applied to the conformational data considered here, the mean of each Gaussian in the selected final computational model corresponds to a preferred χ angle of a nucleoside, and hence, nucleoside rotamer (Fig. S6). The area under each Gaussian is interpreted as the probability of the associated rotamer.

For our data, we observed the most pronounced gain in model quality for models with a low number of components (three to six Gaussians, Fig. S6c). Moreover, each GMM model was examined individually and manually curated if necessary. A model was rejected if it contained two neighbouring peaks, the χ angle values of which were separated by less than 10°. Furthermore, if a selected model contained curves with probabilities lower than 1% and/or standard deviations larger than 50°, the corresponding curve was excluded from the model, and all probabilities were re-calculated based on the remaining curves. The resulting P angle-dependent rotamer library, termed NuConf in the following, contains between three and six rotamers per nucleoside (Table S1). The curves characterizing the + sc peak were manually excluded from the 2´-endo and 3´-endo-like dC model, 2´-endo-like dT model, 3´-endo-like C model, and 3´-endo-like U model according to the above-mentioned cut-off.

In molecular design calculations, computational costs increase significantly with the number of rotamers considered. In order to enable calculations with a smaller conformational search space, we also derived an alternative library with a smaller overall number of rotamers and refer to it as *basic rotamer library* below. To obtain this library, we omitted classifying nucleotides into 2’-endo-like and 3’-endo-like, and fitted single Gaussian curves to each peak in the χ angle distribution of each nucleotide type. This basic P angle-independent library consists of two to three rotamers per nucleoside (Table S1). Similarly to the NuConf library, the final χ angle listings for the nucleotides C, dC, and U are devoid of rotamers describing the + sc conformation.

### Nucleotide-building algorithm

**Fig. 3:**
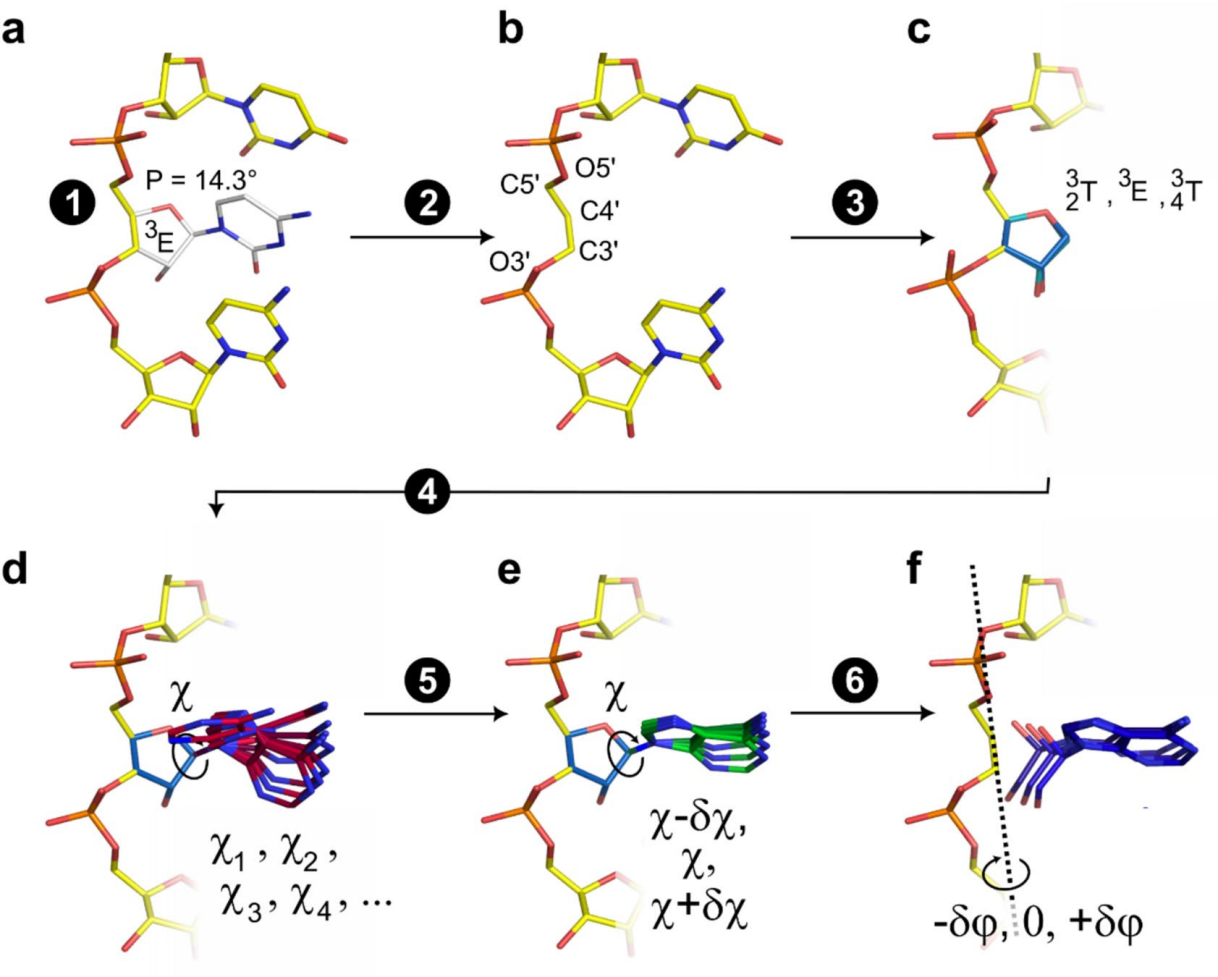
Nucleotide building and generation of nucleoside rotamer diversity with MUMBO. (**a**) Polynucleotide starting template. Evaluation of the pseudorotation angle and ribose/deoxyribose ring puckering at the position of the nucleotide of interest. (**b**) Retention of the nucleotide main-chain atoms and deletion of any additional nucleotide atoms. (**c**) Generation of alternative sugar conformations. (**d**) Nucleobases are added according to the χ angles listed in the nucleoside rotamer library and according to user-specified sequence requests. The rotamers are added to each of the different sugar puckering conformations generated in the previous step. Here, only one of the three previously generated sugar conformations is being displayed. (**e**) Each nucleobase orientation can be further fine-tuned upon addition of user-specified δχ increments. (**f**) Application of a backrub motion resembling that used in protein design in order to further fine-tune the nucleotide orientation^24^. Here, the C3’-C4’ axis is used for the rotation of the coordinates. However, other rotation axes such as the P_i_-P_i+1_ axis can also be chosen.

To assess the quality of the nucleoside rotamer libraries, we implemented the following nucleotide-building routine into the molecular design program MUMBO (Fig. 3). In a first step, P angles are calculated for each nucleotide position of interest. Each position is then classified as being either 2’-endo or 3’-endo-like based on the P angle ranges from above. In a second step, all nucleotide atoms are deleted, except those of the phosphate group and sugar atoms O3’, C3’, C4’, and C5’. These atoms are in the following referred to as nucleotide main-chain atoms.

In a third step, and with the goal to generate a highly diverse set of nucleotide conformations/orientations, several alternative sugar conformations are generated by superposing the coordinates of predefined ribose/deoxyribose molecules onto the main-chain atoms C3’, C4’, and C5’ of the polynucleotide chain provided as input structure. For nucleotide positions identified as 3’-endo-like, three different sugar conformations are added, corresponding to the ring puckering conformations ^3^_2_T, ^3^E, and ^3^_4_T (Fig. 3 and Fig. S1b, d). Similarly, for 2’-endo-like positions, four different ring puckering conformations are added (corresponding to ^2^_3_T, ^2^E, ^2^_1_T, and _1_E) to account for the observation that 2’-endo-like conformations display a broader P angle distribution than 3’-endo-like conformations in case of DNA (Fig. 1a).

In a fourth step, the χ values defining the orientations of the nucleobases are retrieved from the rotamer library, and nucleobases are added to each of the sugar conformations. To further increase the structural diversity provided by the rotamer library, rotamers can be fine-tuned; that is, each rotamer angle χ is expanded by user-defined δχ increments (step 5, Fig. 3). In addition, the so-called backrub motion can be applied. This step reorients an entire nucleotide and introduces some local main-chain flexibility. It conceptionally follows the implementation previously developed for amino acids (step 6, Fig. 3)^24^. In sum, the nucleotide-building procedure generates a highly diverse set of nucleotide/nucleobase orientations at positions of interest.

### Validation of the nucleoside rotamer libraries

To assess how well our nucleoside libraries capture the structural subtleties of nucleic acids, we used MUMBO to computationally rebuild protein side-chain and nucleotide orientations in a dataset derived from experimental structures and determined the agreement between the reconstructed and the experimentally observed coordinates. The dataset was compiled by randomly selecting coordinate spheres from crystal structures subject to certain quality criteria (see methods and materials section, Fig. S7). In particular, we wanted to evaluate which of the two libraries—NuConf or basic library—best reproduces experimentally observed nucleotide conformations. We also analysed how our results are impacted by the application of additional rotamer-building procedures that further expand the structural diversity provided by the rotamer libraries (Table 1). Around 20,000 nucleotides were computationally rebuilt in each of these calculations.

**Table 1:** MUMBO rotamer building performance: How well do nucleoside rotamer libraries reproduce nuclebase orientations in experimental structures?

Run characteristics	Run Number	1	2	3	4	5	6
	Characteristics^a^	NuConf library^b^	Basic library	NuConf library, χ/χ_1_ expansion	Basic library, χ/χ_1_ expansion^c^	NuConf library, backrub motion^d^	NuConf library, χ/χ_1_ expansion, backrub motion
	Average number of initially generated rotamers per nucleotide	32	16	95	49	95	285
	Number of rebuilt nucleotides^e^	20,965	20,061	20,965	20,714	20,943	20,943
Overall RMSD values between closest library-derived orientations and experimentally observed orientations of nucleotides and amino acids (Å)	All nucleotides	0.46	0.56	0.44	0.5	0.35	0.33
	Pyrimidines	0.45	0.48	0.44	0.45	0.35	0.34
	Purines	0.47	0.61	0.44	0.54	0.36	0.32
	Deoxyribonucleotides (dA, dT, dC, dG)	0.51	0.57	0.49	0.52	0.37	0.34
	Ribonucleotides (A, U, C, G)	0.41	0.54	0.39	0.49	0.34	0.31
	Amino acids	0.6	0.6	0.49	0.49	0.54	0.48
Recovery rates—Percentage of matches (%)^f^	All nucleotides	94.6	81.6	97.1	90.6	94.5	97.1
	Deoxyribonucleotides	91.9	76.3	95.8	89.0	91.6	95.5
	Ribonucleotides	97.0	86.9	98.3	92.1	97.3	98.5
	Amino acids	89.2	89.3	94.2	94.2	87.2	91.5

^a^Nucleotides are built using the specified libraries. Amino acids are built using the backbone conformation-dependent library from Dunbrack & Cohen^25^. ^b^NuConf = Gaussian mixture model-derived P angle-dependent library. ^c^Dihedral angle χ in nucleotides and χ_1_ angles in amino acids were expanded by angle increments of − 7, 0 and + 7°. ^d^Nucleotide orientations were further expanded by applying a − 7, 0 and + 7° rotation around the C3’-C4’ axis. ^e^Present in a total of approximately 560 rebuilt spheres. ^f^Matches are postulated based on an analysis of χ angles in nucleotides and χ_1_ angles in amino acids. In case of a match, the corresponding angle in the reference nucleotide/amino acid deviates by less than 15° from that of the library-derived nucleotide/amino acid orientation.

The first conclusion that can be drawn from the data compiled in Table 1 (run #1 *versus* #2) is that the NuConf library performs better than the basic library (RMSD of 0.46 and 0.56 Å, respectively). Note, that with the NuConf library, more rotamers are being built overall than with the basic library; hence, the probability increases that one of the many rotamers contained in the former matches more closely an experimentally observed conformation.

As mentioned above, the conformational space represented by rotamer libraries can be further expanded in MUMBO by adding angle increments (δχ) to each rotamer, by introducing limited local backbone flexibility via the backrub motion algorithm, or by combining these two options. Perhaps unsurprisingly, the best results are obtained with the combined approach, where overall RMSD values of as low as 0.33 Å are reached with the NuConf library (run #6). An increase in prediction accuracy upon introducing angle increments and backbone flexibility is also observed for amino acid side-chains. Strikingly, the NuConf library by itself performs better than the basic library in combination with additional δχ increments (0.46 *versus* 0.50 Å, run #1 *versus* run #4, Table 1).

Overall, nucleotides were modelled more accurately than amino acid side-chains. This is likely due to the planarity of the purine- and pyrimidine ring systems that are less flexible than most amino acids. This rigidity is also the reason why the conformational space of nucleobases can be described by a single dihedral angle—the only parameter that needs to be captured by a computational modelling approach. When considering different types of nucleobases, the data show that pyrimidine- and purine-containing nucleotides are modelled with highly similar accuracies, while the results obtained for ribonucleotides are generally slightly better than for deoxyribonucleotides (Table 1). This holds true irrespective of the library used and the rotamer-expanding mechanism applied.

Apart from the overall RMSD, an additional suitable measure to assess the quality of rotamer-building is the recovery rate, i.e., the percentage of modelled nucleotides whose χ angles fall within, say, ± 15° of the χ angles observed in a reference dataset. In general, recovery rates calculated for our dataset corroborate the conclusions already drawn from the RMSD values reported above (Table 1). The NuConf library scores higher based on this criterion than the basic library, and the best values are obtained upon further expanding the conformational space of the rotamers via the application of δχ increments and backrub motion. Taken together, these data show that the NuConf library clearly outperforms the basic library.

Another important conclusion is that our nucleoside rotamer libraries provide at least the same level of accuracy for constructing nucleotides than well-established amino acid rotamer libraries do for the building of amino acid side-chains.

### Energy-based selection of nucleoside rotamers with MUMBO

While the selection of rotamers based on structural fidelity provides a means to assess how well a rotamer library captures the conformational space adopted by nucleotides in nucleic acids, a more realistic scenario should assess the quality of the side-chain-packing procedure in an unsupervised manner, i.e., when rotamer selection is based solely on the evaluation of interaction energies. MUMBO, like similar protein-design programs, identifies and approximates the energetically most favourable combination of nucleotides and amino acids together with their optimal conformations by applying algorithms based on dead-end elimination and/or Monte Carlo/simulated annealing. The optimal combination is commonly referred to as the *global minimum energy conformation* (GMEC)^4,26^. Using the same reference dataset as described above, we analysed how accurately the energy-derived models capture experimentally observed conformations and determined which program settings produce the best results (Tables 2 and S2). To lower the computational complexity, we reduced the number of nucleotides rebuilt in each optimisation run (to approx. 6700).

**Table 2:** MUMBO rotamer selection performance.

Run characteristics	Run number	1	2	3	4
	Characteristics^a^	NuConf library	Basic library	NuConf library, χ/χ_1_ expansion, backrub motion^b^	NuConf library, χ/χ_1_ expansion, backrub motion, different backrub motion axis^c^
	Number of rebuilt nucleotides^d^	6707	6685	6707	6695
Overall RMSD values between MUMBO-selected orientations of nucleotides and amino acids and orientations observed in reference dataset (Å)	All nucleotides	0.80	1.10	0.65	0.77
	Pyrimidines	0.58	0.65	0.52	0.63
	Purines	0.94	1.34	0.74	0.86
	Deoxyribonucleotides (dA, dT, dC, dG)	0.88	1.17	0.66	0.82
	Ribonucleotides (A, U, C, G)	0.72	1.03	0.65	0.71
	Amino acids	1.73	1.81	1.65	1.66
Recovery rates—Percentage of matches (%)^e^	All nucleotides	85.8	73.7	89.5	86.0
	Deoxyribonucleotides	81.6	69.5	86.6	82.7
	Ribonucleotides	90.4	78.2	92.6	89.5
	Amino acids	78.5	74.2	73.7	73.6
Overall mean per-residue lDDT scores^f^	All in sphere	0.918	0.900	0.925	0.916
	All nucleotides	0.932	0.908	0.946	0.931
	All amino acids	0.910	0.898	0.913	0.909
Overall F_nat_ scores^g^		0.906	0.899	0.917	0.914
Overall INF values^h^		0.866	0.778	0.908	0.889

^a^Nucleotides are built using the specified libraries. Amino acids are built using the backbone conformation-dependent library from Dunbrack & Cohen^25^. ^b^Dihedral angles χ in nucleotides and χ_1_ in amino acids are expanded by − 7, 0 and + 7°. At the same time, nucleotides orientations are further expanded by applying a − 7, 0 and + 7° rotation around the C3’-C4’ axis. ^c^As in run #3, however, in this case, nucleotides orientations were expanded by rotating around the P(i)-P(i + 1) axis. ^d^Present in a total of approx. 600 rebuilt spheres. ^e^Matches are postulated based on an analysis of χ angles in nucleotides and χ_1_ angles in amino acids. In case of a match, the corresponding angle in the reference nucleotide/amino acid deviates by less than 15° from the MUMBO-selected orientation. ^f^Overall mean per-residue lDDT values averaged over all residues/nucleotides and structures within each run^27^. ^g^Fraction of native contacts across nucleic acid–protein interfaces^28^. ^h^Interaction network fidelity values calculated by comparing base-pairing and base-stacking patterns^29^. For a nucleobase-to-nucleobase-specific analysis of the INF values please see Table S2.

The data in Table 2 demonstrate that nucleotide conformations can be predicted reliably with energy-based rotamer selection, and the conclusions drawn in the previous section largely extend to this use case. Thus, the NuConf nucleoside rotamer library performs generally better than the basic library (overall RMSD 0.80 Å *versus* 1.10 Å, run #1 *versus* run #2, Table 2). This is also reflected in the interaction and local fidelity metrics. For instance, the INF values show a marked improvement, indicating a more accurate recapitulation of base-pairing and stacking geometries compared to the basic library (0.866 versus 0.778, Run #1 *versus* #2). In analogy to these findings, the local structural precision, as quantified by the overall mean per-residue lDDT score, follows similar trends. Here, the NuConf library achieves a score of 0.932 for all nucleotides, whereas a value of 0.908 is obtained for the basic library (Table 2). The general quality of the predictions is however lower in this unsupervised approach. Due to the differences in molecular complexity, pyrimidines can be constructed with higher accuracies than purines. While this discrepancy remained irrespective of the type of rotamer library chosen and the mechanism for rotamer-expansion applied, it virtually vanished for the purely structure-based approach described in the previous section, which suggests that the discrimination of rotamers based on interaction energies is more challenging for larger and less symmetric side-chains.

The quality of side-chain placement improves for all nucleotides when the number of starting conformations increases by enabling δχ expansion and backrub motion (RMSD = 0.65 Å *versus* 0.80 Å, run #3 *versus* run #1, Table 2). This improvement is significantly more pronounced for purines (0.74 Å *versus* 0.94 Å, run #3 *versus* run #1, Table 2) than for pyrimidines (0.52 Å *versus* 0.58 Å, run #3 *versus* run #1, Table 2). The recovery rates also increase, albeit to a smaller degree (about 2–5%). This trend is also reflected in the INF and lDDT metrics. Run #3 represents the overall highest prediction accuracy, with INF and nucleotide lDDT values reaching 0.908 and 0.946, respectively (Table 2). Furthermore, the recovery of native contacts at the protein-nucleic acid interface, as assessed by F_nat_ scores, reaches its maximum (0.917, Run #3).

There is a level of freedom of how to define the rotation axis for the backrub motion. We considered two possibilities here, namely an expansion of the nucleotides’ positions by applying a − 7, 0, and + 7° rotation around the C3’-C4’ axis and alternatively around the P(i)-P(i + 1) axis (run #3 *versus* run #4, Table 2). The latter option generally reduced the accuracy with which nucleotide conformations were predicted. The RMSD values increased from 0.65 to 0.77 Å, and the recovery rate decreased from 89.5 to 86% (Table 2). This reduction in performance is also evident in the INF, lDDT, and F_nat_ values (Table 2).

In all cases investigated here, the P-dependent NuConf rotamer library outperforms the basic library. Moreover, the accuracy of the predictions further improves by applying δχ increments and backrub motion^4,24^.

### Comparing the performance of NuConf/MUMBO to similar resources

To further evaluate the performance of the NuConf library in combination with MUMBO, we conducted a comparative benchmarking against two additional modelling tools, namely RNAfitme and MacroMoleculeBuilder (Table 3, Table S3)^30–32^. In terms of coordinate accuracy, MUMBO yields lower RMSD values and Δχ deviations in 6 out of 10 test cases, when compared to RNAfitme. When considering the INF and lDDT values, then MUMBO outperforms RNAfitme in 8 out of 10 structures.

**Table 3:** Comparative modelling performance of MUMBO, RNAfitme, and MacroMoleculeBuilder across a benchmark set of ten reference crystal structures.

PDB ID^a^	Chain	Length	MUMBO				RNAfitme^b^				MacroMoleculeBuilder			
			RMSD (Å)	Δχ(°)	INF^c^	lDDT	RMSD (Å)	Δχ(°)	INF^c^	lDDT	RMSD (Å)	Δχ(°)	INF^c^	lDDT
1CX0	B	72	0.812	10.8	0.860	0.950	0.979	11.5	0.803	0.867	3.096	47.7	0.545	0.731
1EXD	B	73	1.071	16.9	0.778	0.917	1.237	18.6	0.738	0.882	2.040	26.5	0.590	0.840
1FFY	T	75	1.080	14.8	0.868	0.936	1.036	14.2	0.842	0.931	2.075	29.2	0.634	0.839
1GID	A	158	0.896	17.3	0.841	0.931	0.803	15.8	0.799	0.872	2.292	27.4	0.652	0.832
1I6U	C	37	0.540	5.3	0.913	0.957	1.097	12.7	0.865	0.912	1.851	33.9	0.662	0.823
1MMS	C	58	0.467	4.4	0.946	0.972	0.753	14.2	0.814	0.904	2.389	30.6	0.629	0.818
1U0B	A	74	0.652	8.0	0.849	0.947	0.719	9.0	0.839	0.930	1.783	26.5	0.711	0.872
1UN6	E	61	0.639	9.6	0.919	0.960	0.950	16.3	0.844	0.898	1.817	26.6	0.653	0.852
1WZ2	C	88	1.244	20.6	0.696	0.889	1.240	17.3	0.747	0.891	2.107	23.8	0.667	0.843
1Y0Q	A	229	1.120	19.3	0.717	0.892	0.919	15.2	0.766	0.893	1.693	20.4	0.692	0.870

^a^The reference structures were taken from Antczak et al.^30^. As judged from sequence identity comparisons, all entries, except for entries 1FFY and 1U0B, were not part of the dataset from which the NuConf library was derived. Therefore, these structures provide for an opportunity of an independent cross-validation. ^b^The RNAfitme results shown here appear slightly poorer than those reported by Antczak et al.^30^. This is because RNAfitme was used without a final energy minimization step. This was done for comparison reasons since such a step is currently not implemented in MUMBO. ^c^For a nucleobase-to-nucleobase-specific analysis of the INF values please see Table S3.

Both MUMBO and RNAfitme consistently yield better results than program MacroMoleculeBuilder, when considering all four validation indicators and across all 10 test cases. Of all test calculations, the lowest RMSD (i.e. 0.47 Å) and Δχ (4.4°) values and highest INF (0.946) and lDDT (0.972) scores were obtained for pdb entry 1MMS with program MUMBO (Table 3). These results show that the NuConf library in combination with MUMBO allows for an accurate recapitulation of complex RNA architectures and interaction networks.

### Modelling of nucleic acids and protein-nucleic acid interfaces with MUMBO

Having assessed the quality of the rotamer libraries, we applied our nucleic acid design approach to the problem of repacking interfaces within a DNA duplex and a protein-RNA complex, thereby assessing the performance of our implementation in a relevant molecular modelling scenario. Hallmarks of the structural biology of nucleic acids are base pair complementarity and protein-interaction specificity. Modelling these interactions with sufficient accuracy constitutes a prerequisite for the design of new systems.

**Fig. 4:**
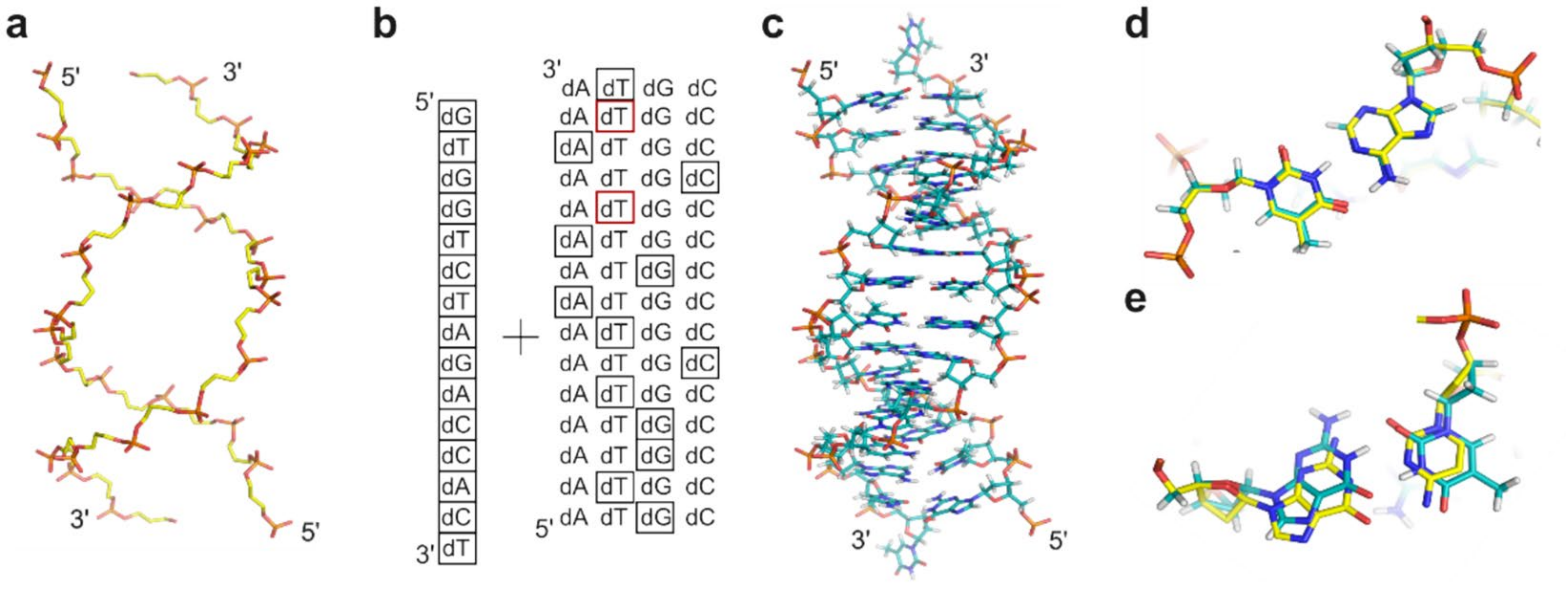
Base pair complementation with MUMBO. (**a**) Starting DNA backbone taken from PDB entry 4U0Y^33^. (**b**) MUMBO sequence input and sequence selection result. While for one strand a defined sequence was provided as input, all four types of deoxynucleotides were offered as input for the second strand. Boxed nucleotides show the results of the energy-driven selection process. Of the 14 base pairs present in 4U0Y, 12 base pairs are predicted correctly (black boxes, 86%) and 2 incorrectly (red boxes). (**c**) MUMBO-generated structure. (**d**) Superposition of MUMBO-predicted dT-dA base pair (in cyan) and base pair as observed in 4UOY (in yello0w). (**e**) Wrongly predicted dG-dT base pair (in cyan) in comparison to the dG-dC base pair observed in 4UOY (in yellow).

In a first example, the structure of a palindromic double-stranded DNA fragment was considered (PDB entry 4U0Y)^33^. We were interested in the question whether energy-driven nucleotide selection as implemented in MUMBO can reproduce the base pair complementarity in a DNA fragment. To this end, we rebuilt both strands of a DNA fragment (the starting backbone structure of which is shown in Fig. 4a) as follows: For one strand, the native sequence as observed in the crystal structure was provided as input, but MUMBO was asked to select optimal nucleobase orientations. For the complementary strand, however, the program had to select both the identity of each nucleotide to be built and its spatial orientation. As shown in Fig. 4b, c, MUMBO predicted the identities of 12 out of 14 base pairs correctly and captured their conformations with high accuracy (RMSD = 0.42 Å, averaged deviation in χ = 6.2°). The comparison of the predicted and the reference structure (Fig. 4d, e) shows that while the correctly predicted complementary nucleobases are in good structural agreement with the experimental template, wrong predictions result in larger structural deviations.

**Fig. 5:**
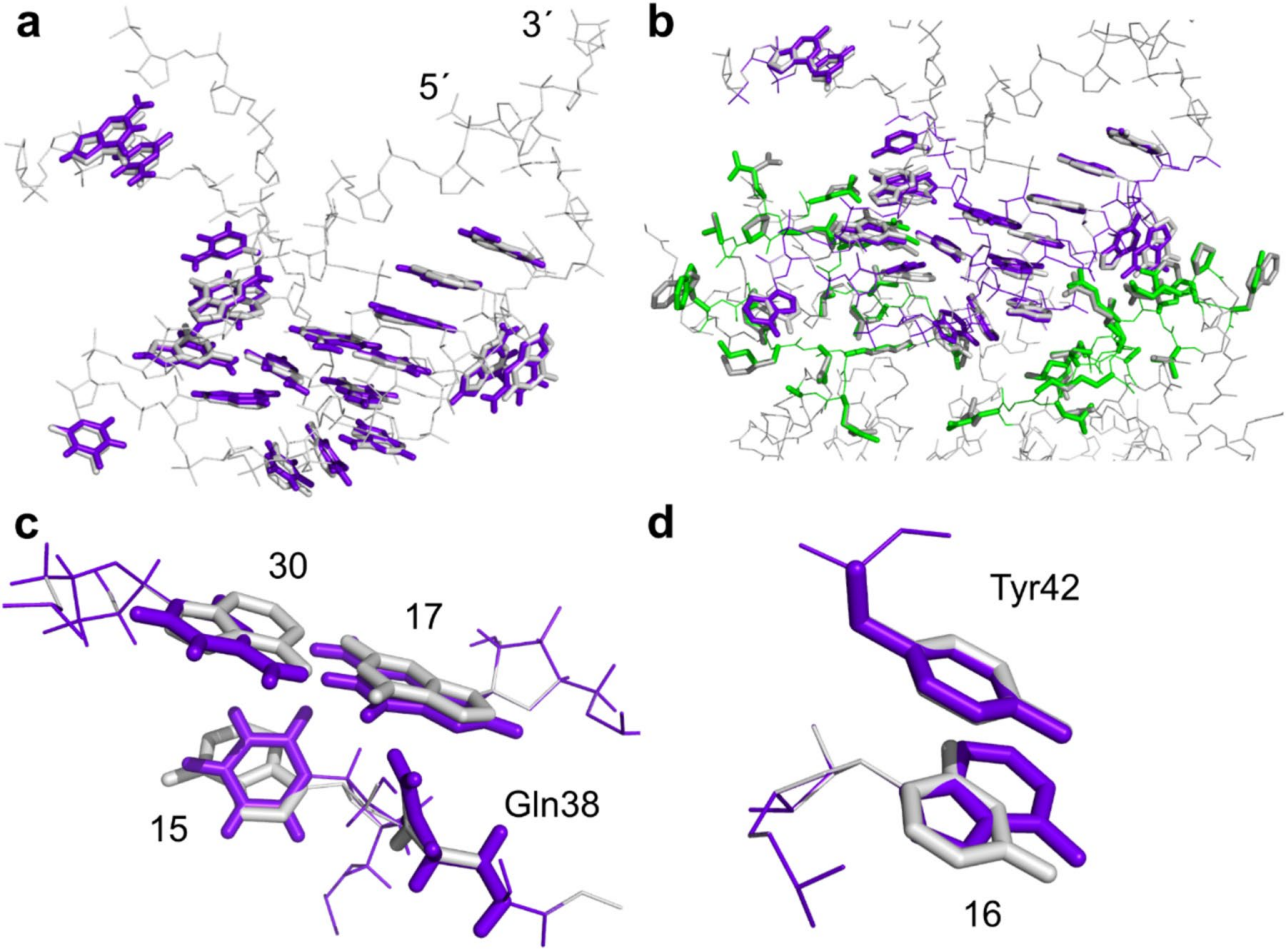
Rebuilding a protein-RNA interface with MUMBO. (**a**) Superposition of the RNA aptamer input structure (PDB entry 6SY4^34^) with a MUMBO-generated output structure obtained after rebuilding the aptamer-protein interface with the original sequence as input. Only nucleobases located within the protein-RNA interface are shown. (**b**) MUMBO-derived model obtained after allowing for RNA sequence variability and comparison of the model with the original input structure. (**c**) Example of a MUMBO-introduced nucleobase substitution. Nucleotide A30 in the original structure is exchanged to cytidine thereby introducing a canonical base pair C30-G17 instead of the original non-canonical base pair A30-G17. At the same time, the sequence-specific interaction between G17 and Gln38 is retained. (**d**) MUMBO proposes that a substitution of U16 against adenine would be energetically advantageous. In case of both U16 and A16, a π-stacking interaction with Tyr42 is retained. In all panels, the original crystal structure is shown in grey and the MUMBO-derived models in purple and green.

In order to investigate how well MUMBO predicts protein-nucleic acid interactions, we selected the crystal structure of the transcriptional repressor TetR in complex with the RNA aptamer K1 as a second example (PDB entry 6SY4^34^) . In particular, we tasked the program with rebuilding the TetR/aptamer interface and focused on 20 nucleobases and 36 amino acids located within 7 Å of the interface of the complex. In a first step, we determined the quality of nucleoside rotamer selection in the interface region using the native RNA and protein sequence as input. Very encouragingly, MUMBO predicted all nucleobase orientations with high structural fidelity and without any steric clashes (RMSD = 0.42, average χ deviation = 5.9°) (Fig. 5a). Next, we probed the design capabilities of our approach and let the program select the energetically most favourable type of nucleotide at each of the 20 nucleotide positions. MUMBO modelled 15 out of 20 nucleobases with the original sequence (Fig. 5b). The selected conformations are very close to those observed in the crystal structure (RMSD = 0.40 Å, average χ deviation = 5.0°). Five nucleotides are replaced by other nucleotides at positions involved in base pairings but also at unpaired positions (Fig. 5c, d). Thus, the non-canonical base pair A30-G17 is replaced by a canonical base pair C30-G17. We observe that also in the case of substituted nucleotides, protein interactions similar to those seen in the original structure are retained. Whether the substitutions proposed by MUMBO improve aptamer stability and/or protein-binding affinities in vivo will have to await further experimental validation.

## Discussion

In this work, we demonstrate that the simultaneous and seamless modelling of amino-acid and nucleic-acid side-chains within the same computational framework—and hence the computational design of protein/nucleic acid interfaces—can be achieved by extending the concept of rotamer libraries commonly used in protein design to nucleic acids. Rotamer libraries have proven to be invaluable tools to reduce computational complexity in the field of computational protein design. They map the conformational space theoretically available to side-chains to a finite and hence enumerable set and make the computational problem amenable to discrete optimization algorithms.

The statistical analysis of the dataset we compiled reveals some interesting static and dynamic principles of nucleic acids. The pseudorotation angle (P) distribution, which describes the conformations of the sugar moiety, is dominated by two populations, which can be ascribed to the 2’-endo (preferred in DNA) and 3’-endo ribose/deoxyribose puckering (preferred in RNA) (Fig. 1a, b). Moreover, we observe significantly broader distributions for the respective P values for DNA in comparison to RNA. Thus, the peak describing the preferred 3’-endo conformation in RNA is significantly narrower than the 2’-endo peak in the case of DNA and this also holds true for the direct comparison of the 3’-endo peaks in both RNA and DNA. This shows that within the constraints of their respective biopolymers, ribose can access neighbouring puckering conformations less readily than deoxyribose, presumably caused by the steric demands of the 2’-OH group in the ribose moiety. Likewise, the involvement of the ribose 2’-OH group in hydrogen-bonding networks might also restrict the ribose ring puckering.

Similar differences in structural variability can be observed for the distributions of the nucleoside-specific rotamer angle χ (Fig. 1c, d). Anti-like orientations, which facilitate A and B double helix formation, are by far the most common conformation. Yet again, the RNA-specific –ap conformations displays a considerably narrower distribution than the same conformation in DNA or the DNA-specific –ac conformation.

Our data also show that the pseudorotation angle P and the side-chain angle χ are strongly correlated in both RNA and DNA (Fig. 2). Thus, the 2’-endo ring puckering conformation is preferred in association with the –ac nucleobase orientation and the 3’-endo conformation with the –ap nucleobase orientation. But also in this case, the more pronounced narrowness (and concomitant separability) of the RNA-specific distributions lead to a clearer delineation of the angular data into two individual populations compared to the DNA-related data. Importantly, this correlation, together with the distinct clustering of the χ angle data, allowed us to derive the backbone-dependent NuConf rotamer library with improved structural-modelling capabilities in comparison to a basic rotamer library that does not factor in this correlation.

In the field of statistical modelling, overfitting of model parameters is a general concern, and in the NuConf library, we strived to represent the conformational space with the smallest number of rotamers that still fully captures the information content of the underlying dataset. Our validation calculations with MUMBO showed that the NuConf library is able to reproduce the nucleotide conformations observed in experimentally determined crystal structures quite reliably. When comparing the performance of NuConf/MUMBO to that of similar programs, we observed that MUMBO performs slightly better than RNAfitme and considerably better than MacroMoleculeBuilder^30,31^. A major advantage of MUMBO in comparison to RNAfitme is that MUMBO is a sequence design tool that allows to explore and optimize alternative sequences for both DNA and RNA polynucleotides as well as for protein-DNA and protein-RNA interfaces. In its current version, RNAfitme is limited to the modelling of RNA molecules starting from a single input sequence^30^.

MUMBO and RNAfitme outperformed MacroMoleculeBuilder. This was unexpected since MacroMoleculeBuilder was used in a mode that did not require the program to combinatorically identify the best nucleotide conformation from a library containing multiple conformations. Instead, MacroMoleculeBuilder was asked to rebuild the structures using NtC dinucleotides that matched the conformation of the dinucleotides from the input structure. Moreover, base-pairing information was also provided as input. Yet, the resulting INF scores that quantify differences in nucleotide packing and base pairing between model and reference structure were consistently poorer than with MUMBO and RNAfitme, which were not provided with this additional input information.

We conclude that the NuConf/MUMBO approach of using dedicated nucleoside rotamer libraries in combination with side-chain-packing algorithms generates models of nucleic acids and their complexes with proteins that closely resemble native geometries and captures the details of canonical and non-canonical base pairing with high accuracy.

## Methods

### Compilation of reference datasets

Two different reference datasets were compiled. Reference dataset one was generated for the statistical analysis of nucleotide geometries (Table S4). Structures describing either RNA, DNA, RNA–protein or DNA–protein complexes solved by X-ray crystallography with resolutions better than 2.5 Å and released between 2010 and 2021 were identified using the nucleic acid knowledgebase resource/nucleic acid database and the entries downloaded from the protein data bank^35,36^. The dataset also contains some entries originally published before 2010. In these cases, a revised and updated version of the entry was published within the 2010 to 2021-time span. A total of 4518 entries containing 176,441 nucleotides were retrieved (Table S4).

A second reference dataset was compiled to validate (i) the quality of the derived nucleotide libraries and (ii) the nucleotide selection algorithms implemented in MUMBO. This dataset consists of 4445 atom coordinate spheres with radii of 30 Å and containing snapshots of either protein-DNA or protein-RNA complexes (Table S5). These coordinate sphere files were created as follows: Firstly, crystal structures solved at resolutions better than 2.0 Å and released before February 2023 containing either DNA–protein (1589 structures) or RNA–protein complexes (446) were retrieved from the protein data bank (Fig. S7)^35^. Subsequently, structure duplicates were eliminated upon inspection of cell axes and angles of the crystals used for the crystal structure determination. Atom coordinate spheres were then generated from these structures by selecting every 5th phosphor atom present in the nucleotide part of the structure as a sphere centre and by including all atoms present within 30 Å distance of the sphere centre into an atom coordinate sphere file. Crystallographic symmetry (and hence crystal packing) was also accounted for in order to include all relevant interactions present within the spheres in the underlying crystal structures. Therefore, the atomic coordinates of symmetry-related residues were added to an individual coordinate sphere if any symmetry-related atoms were located within 30 Å of the spheres’ centre (Fig. S7). Please note that for the validation calculations, only data subsets consisting of approximately 600 coordinate spheres were used.

Neither during the compilation of the first nor the second reference dataset, a sequence identity or structure homology cut-off was applied.

### Statistical analysis of nucleobase geometry

﻿χ angle and pseudorotation angle (P) values were analysed for all nucleotides present in reference dataset one (Table S4). χ angles were calculated from the positions of atoms O4’, C1’, N9 and C4 for purine nucleotides and atoms O4’, C1’, N1 and C2 for pyrimidines. The pseudorotation angle P was calculated from the five torsion angles ν_0_, ν_1_, ν_2_, ν_3_ and ν_4_ that describe the puckering of the furanose ring in each nucleotide and using the formula proposed by Altona and Sundralingam (Fig. S1a)^16^. Please note that in order to generate P values covering the entire 360° range and to distinguish between ‘mirrored’ conformations (i.e., for example, ^1^_2_T *versus* ^2^_1_T), 180° were added to the calculated P value in case of negative ν_2_ values^16^. Pseudorotation amplitudes (τ_M_) were calculated using the formalism proposed by Rao et al.^17^.

### Nucleoside rotamer library generation using Gaussian mixture models

The Gaussian mixture model (GMM) for an underlying univariate distribution is represented by a linear combination of Gaussian functions (or components, where each component has a weight of 1) describing the amount of data points from the mixed distribution that belongs to the component (Fig. S6). To generate a mixture model, arbitrary initial values for mean, variance, and weight are assigned to each component for a user-defined number of components. This statistical model is then fit to data by means of the two-step Expectation–Maximization algorithm^21,37^. The expectation step includes evaluation of a variable called responsibility for each data point in the distribution based on the given parameters. The responsibility is defined as the posterior probability of a data point being a part of a component in the model. The maximization step uses the obtained responsibilities to recalculate means, variances and weights. These two steps are repeated until the estimation of the log-likelihood of the maximization step converges^21^. The quality of the resulting model is assessed by calculating the Bayesian information criterion: *BIC* = − 2ln[L(M)] + *k*ln*N*, where *L* is the maximized value of the likelihood function of a model *M*, *k* is the number of parameters estimated by a model, and *N* is the number of data points. The model that produces the best fit for the underlying data is indicated by the first local minimum of the estimator^23^.

We used the Python package sklearn.mixture for generating Gaussian mixture models and calculating the Bayesian information criterion^38^. As domain for our dataset, we selected the angular range [0°, 360°). Due to the absence of peaks in the 0°/360° interval boundary region, we were able to apply GMM to our data directly, without the necessity to consider circular statistics. We interpreted mean values of the Gaussian components as preferred χ angle values of rotamers, and their respective weights as rotamer probabilities. Prior to the calculations of nucleotide-specific GMM models, the nucleotides were further subdivided into 3´-endo-like and 2´-endo-like classes (Fig. S6, Table S6).

To identify the optimal number of fitted Gaussian curves, GMMs containing between 1 and 20 curves were generated for each χ angle distribution, and a Bayesian information criterion score was calculated for each model (Fig. S6c). The first local minima were typically observed in models containing 3–5 components. The output parameters (mean values, variances, and probabilities) of these models and neighboring ones were examined and manually curated, if necessary. Thus, models containing Gaussian components with differences in mean values smaller than 10° were rejected. If the selected model contained a curve with probabilities smaller than 1%, a curve with standard deviation higher than 50° or both, then these curves were excluded from the model, and the probabilities for the remaining curves recalculated. The mean values of the final models were rounded to integers and together with their respective probabilities assembled into a P angle-dependent rotamer library, termed NuConf.

### Basic nucleoside rotamer library compilation

In order to construct a simpler and more basic library, single Gaussian curves were fitted to manually identified peaks in user-specified intervals within the χ angle distributions by means of a Python script. The mean value of the fitted peak was interpreted as the χ angle of the rotamer. The area under the fitted curve in proportion to the sum of all curve areas fitted was used to calculate the respective rotamer probabilities (Fig. S6d).

### Quantification of the rotamer library performances

We used the MUMBO program to validate the nucleoside rotamer libraries and compared MUMBO-generated and library-derived nucleotide conformations to the nucleotide conformations present in the original structures. Nucleotides were built in MUMBO as outlined (Fig. 3). As reference datasets, we used an equal number of protein-DNA or protein-RNA atom coordinate spheres (Table S5 and Fig. S7). In a first step, we generated multiple amino acids and nucleotide conformations for all protein side-chains and nucleotides with atoms located within 25 Å of the sphere center (Fig. S8). Amino acids and nucleotides located outside of this radius were left unaltered. On average, about 150 amino acid side-chains and 35 nucleotide positions were rebuilt in each atom coordinate sphere. For library validation, we subsequently inspected all MUMBO-generated protein side-chain and nucleotide orientations and identified those that resembled the closest the conformations present in the native structure based on the lowest RMSD value between any MUMBO-generated conformation and the conformation present in the original structure. This was achieved using the PICKNAT module in MUMBO. Please note that the number of alternative nucleotide conformations being built at a specific position corresponds to the number of χ angle entries in the corresponding rotamer library multiplied by (i) the number of alternative sugar conformations considered at this position and (ii) the number of alternative H-atom placements in nucleotide H-bond donor atoms. This number is further multiplied by three in case χ angles are expanded by adding -δχ, 0 and + δχ increments to each χ angle. The same holds true in case a backrub motion is applied. These calculations were repeated using two different nucleoside rotamer libraries.

### Validation of the nucleotide and protein side-chain selection algorithms implemented in MUMBO

To validate the performance of the energy-driven selection process as implemented in MUMBO, we performed calculations similar to those used to quantify the quality of different rotamer libraries and made again use of the protein-DNA or protein-RNA atom coordinate sphere reference data (Table S5 and Fig. S7). However, instead of merely picking those orientations that compared best to the orientations present in the reference datasets, the selection was now energy-driven through the calculation of interatomic interaction energies and using the various side-chain-packing algorithms implemented in MUMBO. In order to do so, we considerably altered MUMBO so that MUMBO is now able to simultaneously identify the energetically most favourable protein side-chain conformations and nucleotide orientations in protein-RNA or protein-DNA complexes.

In contrast to the calculations performed for validating the nucleoside rotamer libraries, nucleotides and amino acids were rebuilt if located within 12 instead of 25 Å of the sphere centres. On average, 10 nucleotides and 15 amino acid residues were rebuilt in each atom coordinate sphere. The results from these calculations were statistically analysed using procedures similar to those used for nucleotide rotamer library validation and as described above.

In addition to the above validation calculations, we also tested the performance of MUMBO using selected examples of specific protein-DNA or protein-RNA interaction complexes. For assessing the quality of base pair complementation and DNA-fragment reconstruction by MUMBO, we selected as an example the crystal structure of the bacterial repressor NagR in complex with a fragment of its operator sequence (PDB entry 4U0Y)^33^. To study the fidelity with which protein-nucleic acid interactions are captured by MUMBO, we chose the crystal structure of the bacterial repressor TetR in complex with a specific RNA aptamer (PDB entry 6SY4^34^) and considered all nucleobases within 7 Å from the protein/RNA interface in repacking calculations. For each calculation, we enabled χ-expansion and backrub motion with an angular increment of 7 degrees.

### Comparative modelling and benchmarking

The performance of NuConf/MUMBO was compared to that of programs RNAfitme and MacroMoleculeBuilder 3.0, using a representative set of ten reference structures (Table 3)^30–32^. For RNAfitme, we used the Neural gas-Euclidean-high-resolution (NG-E-HR) nucleotide conformer library with the nucleoside residue remodeling option enabled^30^. Energy minimization of the final model was disabled in RNAfitme to allow a direct comparison with MUMBO, which does not include a final energy minimization step.

MacroMoleculeBuilder calculations were performed with the polynucleotide backbone as input. As additional input restraints, the input reference structure was matched with single NtC dinucleotides using the DNATCO platform and base-pair interaction restraints were derived from the reference structure using the RNA-Puzzles assessment toolkit^12,39,40^. This differs from the use of programs RNAfitme and MUMBO, which were executed without any prior input information on base stacking and base pairing. The backbone was kept rigid by applying the “Mobilizer Rigid” command in MacroMoleculeBuilder. To ensure a focused refinement, the degrees of freedom were strictly restricted to the C1′–N1/N9 glycosidic bonds and the C4′–C1′ and C3′–C2′ sugar linkages. All calculations were executed using the Amber99 force field, and local steric clashes were addressed using the “Periodic Scrubber” mechanism^41^.

### Performance indicators for structure comparisons

The obtained models and reference structures were compared using multiple performance indicators. Overall RMSD values were calculated by including all atom position deviations in a single RMSD value calculation. Alternatively, average RMSD values could also have been reported. In the case of the latter, RMSD values are calculated for each compared nucleotide/amino acid separately and subsequently the average RMSD value is deduced. We observed that the latter values would have been about 20 to 40% lower than the overall RMSD values reported in the present study. The following atoms were excluded from the RMSD calculations: P, OP1, OP2, O5’, C5’, C4’, C3’, O3’ in case of nucleotides and N, CA, C, O and CB in case of amino acids. Recovery rates report the percentage of χ angle matches. A match is postulated in case the χ angles in nucleotides and χ_1_ angles in amino acids in the reference nucleotide/amino acid deviates by less than 15° from the MUMBO-generated orientation. In case of nucleotides, Δχ deviations report the average χ angle difference between model and reference structure.

The structural fidelity of key interactions was quantified by additional assessment metrics. Interaction network fidelity (INF) values were determined following the definition of Parisien et al.^29^. Base-pairing and base-stacking true positives (TP), false negatives (FN), and false positives (FP) were identified for each individual structure using the RNA-Puzzles assessment toolkit^40^. Overall INF values were then computed for each validation run based on the total number of TP, FN, and FP. Local structural accuracy was further quantified using the local distance difference test (lDDT)^27^. Per-residue lDDT scores were computed via OpenStructure and subsequently averaged to yield a mean score for each structure^42^. Subsequently, these values were averaged across all structures within a given run to derive an overall mean per-residue lDDT. In instances involving protein–nucleic acid interfaces, the fraction of native contacts (F_nat_) was additionally determined to monitor whether the interfaces were accurately recapitulated within our modelling framework^42,28^.

### Software used for data processing and visualization

All structure representations were illustrated using PyMOL^43^. Data processing, analysis and illustration was done using Python, R, and FORTRAN 95 scripts/programs^44–46^. The reference datasets were compiled using C-shell and Python scripts, the CCP4 crystallographic software package and in-house computer programs^47^. Scripts and complete lists of all protein data bank entries used in the present study are available at zenodo.org (see data availability statement).

## Supplementary Information

Supplementary Information.

## Data Availability

The nucleoside rotamer library and the rebuilding of DNA/RNA–protein interfaces is implemented in the latest version of computer program MUMBO. Program MUMBO and all additional computer scripts, computer programs and datasets (including 4445 coordinate spheres) used in the present study have been deposited with zenodo.org (https://zenodo.org/records/18431987). Program MUMBO is also available from the software repository Gitlab (https://gitlab.com/group_muller/mumbo_software3).
