# Supplementary material for: NuConf: a rotamer library for DNA and RNA and its implementation in the protein design software MUMBO
Source: Sci Rep. 2026 May 26;16:16281. doi: 10.1038/s41598-026-52380-3 (PMC13212916; doi:10.1038/s41598-026-52380-3)
Supplement: Supplementary file 1 — Supplementary Information. [file 41598_2026_52380_MOESM1_ESM.docx]

**Supplementary Information**

**NuConf - A rotamer library for DNA and RNA**

**and its implementation in the protein design software MUMBO**

**Marharyta O. Makarova^1^, Martin T. Stiebritz^1^, Derman Basturk^1,2^, Birthe Lemke^1^, Beatrix Süss^3,4^ and Yves A. Muller^1^**

^1^Division of Biotechnology, Department of Biology, Friedrich-Alexander-Universität Erlangen-Nürnberg (FAU), 91052 Erlangen, Germany.

^2^Max Planck Center for Physics and Medicine, 91054 Erlangen, Germany

^3^Department of Biology, Technische Universität Darmstadt, 64287 Darmstadt, Germany

^4^Centre for Synthetic Biology, Technische Universität Darmstadt, 64287 Darmstadt, Germany.

|  | **NuConf library** | | | | **Basic library** | | |
| --- | --- | --- | --- | --- | --- | --- | --- |
| **Nucleobase** | **P angle** | **Mean, [°]** | **σ, [°]** | **Probability** | **Mean, [°]** | **σ, [°]** | **Probability** |
| **A** | **2´-endo** | 66 | 15.0 | 0.101 | 65 | 11.2 | 0.038 |
|  |  | 200 | 23.8 | 0.063 | 198 | 8.4 | 0.962 |
|  |  | 221 | 9.0 | 0.246 |  |  |  |
|  |  | 243 | 8.1 | 0.250 |  |  |  |
|  |  | 262 | 11.5 | 0.157 |  |  |  |
|  |  | 284 | 16.9 | 0.182 |  |  |  |
|  | **3´-endo** | 65 | 30.8 | 0.019 |  |  |  |
|  |  | 185 | 8.6 | 0.194 |  |  |  |
|  |  | 197 | 5.2 | 0.509 |  |  |  |
|  |  | 208 | 6.5 | 0.180 |  |  |  |
|  |  | 228 | 9.1 | 0.055 |  |  |  |
|  |  | 261 | 21.4 | 0.043 |  |  |  |
| **C** | **2´-endo** | 65 | 21.5 | 0.046 | 199 | 5.7 | 0.902 |
|  |  | 204 | 14.9 | 0.105 | 229 | 19.8 | 0.098 |
|  |  | 229 | 8.0 | 0.406 |  |  |  |
|  |  | 246 | 7.3 | 0.316 |  |  |  |
|  |  | 277 | 19.6 | 0.128 |  |  |  |
|  | **3´-endo** | 192 | 6.3 | 0.216 |  |  |  |
|  |  | 199 | 3.9 | 0.558 |  |  |  |
|  |  | 206 | 5.1 | 0.198 |  |  |  |
|  |  | 218 | 23.6 | 0.028 |  |  |  |
| **G** | **2´-endo** | 67 | 13.6 | 0.118 | 69 | 8.1 | 0.022 |
|  |  | 198 | 22.0 | 0.071 | 194 | 7.2 | 0.874 |
|  |  | 230 | 8.1 | 0.175 | 250 | 21.5 | 0.104 |
|  |  | 246 | 8.2 | 0.263 |  |  |  |
|  |  | 263 | 8.1 | 0.243 |  |  |  |
|  |  | 285 | 14.2 | 0.129 |  |  |  |
|  | **3´-endo** | 58 | 35.2 | 0.018 |  |  |  |
| continued on the next page | | | | | | | |
|  | **NuConf library** | | | | **Basic library** | | |
| **Nucleobase** | **P angle** | **Mean, [°]** | **σ, [°]** | **Probability** | **Mean, [°]** | **σ, [°]** | **Probability** |
| **G** | **3´-endo** | 186 | 6.6 | 0.284 |  |  |  |
|  |  | 195 | 5.0 | 0.535 |  |  |  |
|  |  | 207 | 8.0 | 0.132 |  |  |  |
|  |  | 247 | 25.6 | 0.032 |  |  |  |
| **U** | **2´-endo** | 58 | 25.2 | 0.018 | 200 | 10.5 | 0.714 |
|  |  | 201 | 15.7 | 0.072 | 228 | 18.2 | 0.286 |
|  |  | 226 | 8.4 | 0.386 |  |  |  |
|  |  | 242 | 8.6 | 0.428 |  |  |  |
|  |  | 268 | 24.7 | 0.095 |  |  |  |
|  | **3´-endo** | 194 | 7.8 | 0.248 |  |  |  |
|  |  | 201 | 5.0 | 0.651 |  |  |  |
|  |  | 217 | 11.5 | 0.086 |  |  |  |
|  |  | 260 | 33.6 | 0.015 |  |  |  |
| **dA** | **2´-endo** | 68 | 19.2 | 0.020 | 65 | 14.1 | 0.021 |
|  |  | 222 | 21.3 | 0.064 | 206 | 15.8 | 0.153 |
|  |  | 244 | 8.6 | 0.235 | 254 | 11.6 | 0.826 |
|  |  | 257 | 7.7 | 0.473 |  |  |  |
|  |  | 266 | 16.2 | 0.207 |  |  |  |
|  | **3´-endo** | 64 | 29.3 | 0.039 |  |  |  |
|  |  | 190 | 8.2 | 0.237 |  |  |  |
|  |  | 205 | 7.5 | 0.399 |  |  |  |
|  |  | 220 | 11.0 | 0.136 |  |  |  |
|  |  | 245 | 20.9 | 0.190 |  |  |  |
| **dC** | **2´-endo** | 211 | 11.8 | 0.110 | 205 | 9.8 | 0.230 |
|  |  | 238 | 7.5 | 0.354 | 245 | 16.8 | 0.770 |
|  |  | 253 | 8.0 | 0.335 |  |  |  |
|  |  | 270 | 11.9 | 0.181 |  |  |  |
|  | **3´-endo** | 200 | 7.6 | 0.548 |  |  |  |
| continued on the next page | | | | | | | |
|  | **NuConf library** | | | | **Basic library** | | |
| **Nucleobase** | **P angle** | **Mean, [°]** | **σ, [°]** | **Probability** | **Mean, [°]** | **σ, [°]** | **Probability** |
| **dC** | **3´-endo** | 219 | 9.3 | 0.318 |  |  |  |
|  |  | 240 | 14.6 | 0.134 |  |  |  |
| **dG** | **2´-endo** | 67 | 9.6 | 0.035 | 65 | 7.8 | 0.047 |
|  |  | 232 | 21.1 | 0.096 | 196 | 10.5 | 0.154 |
|  |  | 245 | 7.9 | 0.304 | 255 | 13.3 | 0.799 |
|  |  | 259 | 7.0 | 0.375 |  |  |  |
|  |  | 271 | 10.3 | 0.190 |  |  |  |
|  | **3´-endo** | 62 | 9.2 | 0.109 |  |  |  |
|  |  | 192 | 8.1 | 0.520 |  |  |  |
|  |  | 207 | 10.3 | 0.244 |  |  |  |
|  |  | 242 | 13.6 | 0.105 |  |  |  |
|  |  | 287 | 18.9 | 0.021 |  |  |  |
| **dT** | **2´-endo** | 217 | 21.3 | 0.078 | 65 | 15.8 | 0.011 |
|  |  | 244 | 9.2 | 0.602 | 207 | 14.5 | 0.148 |
|  |  | 256 | 16.3 | 0.320 | 245 | 12.5 | 0.841 |
|  | **3´-endo** | 71 | 25.4 | 0.025 |  |  |  |
|  |  | 200 | 10.8 | 0.474 |  |  |  |
|  |  | 222 | 15.5 | 0.367 |  |  |  |
|  |  | 239 | 33.0 | 0.134 |  |  |  |

# Table S1. P angle-dependent NuConf rotamer library and P angle-independent basic rotamer library.

| Run characteristics^a^ | Run number | | 1 | 2 | 3 | 4 |
| --- | --- | --- | --- | --- | --- | --- |
|  | Run characteristics | | NuConf library | Basic library | NuConf library, χ/χ1 expansion, backrub motion | NuConf library, χ/χ1 expansion, backrub motion, different backrub motion axis |
| Base pair-specific INF values | Stacking | Upward | 0.891 | 0.839 | 0.917 | 0.905 |
|  |  | Downward | 0.641 | 0.409 | 0.772 | 0.693 |
|  |  | Inward | 0.528 | 0.266 | 0.660 | 0.628 |
|  |  | Outward | 0.742 | 0.460 | 0.799 | 0.776 |
|  |  | Total | 0.868 | 0.779 | 0.901 | 0.886 |
|  | Watson Crick-Watson Crick | Cis | 0.893 | 0.826 | 0.944 | 0.923 |
|  |  | Trans | 0.566 | 0.074 | 0.526 | 0.351 |
|  |  | Total | 0.892 | 0.820 | 0.942 | 0.920 |
|  | Hoogsteen-Hoogsteen | Cis | 0.408 | 0.000 | 0.408 | 0.316 |
|  |  | Trans | 0.258 | 0.671 | 0.775 | 0.224 |
|  |  | Total | 0.309 | 0.378 | 0.617 | 0.252 |
|  | Sugar Edge-Sugar Edge | Cis | 0.000 | 0.000 | 0.000 | 0.000 |
|  |  | Trans | 0.853 | 0.603 | 0.746 | 0.798 |
|  |  | Total | 0.763 | 0.539 | 0.746 | 0.746 |
|  | Watson Crick-Hoogsteen | Cis | 0.328 | 0.228 | 0.543 | 0.454 |
|  |  | Trans | 0.640 | 0.350 | 0.790 | 0.743 |
|  |  | Total | 0.457 | 0.238 | 0.664 | 0.587 |
|  | Watson Crick-Sugar Edge | Cis | 0.404 | 0.143 | 0.382 | 0.196 |
|  |  | Trans | 0.548 | 0.408 | 0.791 | 0.685 |
|  |  | Total | 0.474 | 0.287 | 0.629 | 0.467 |
|  | Hoogsteen-Sugar Edge | Cis | 0.640 | 0.175 | 0.647 | 0.640 |
|  |  | Trans | 0.888 | 0.744 | 0.923 | 0.866 |
|  |  | Total | 0.832 | 0.607 | 0.868 | 0.816 |

# Table S2. Base pair-specific details of the INF values reported in Table 2 of the main text.

|  | PDB ID | Chain | Base pair specific INF values | | | | | | | | | | | | | | | | | | | | | | |
| --- | --- | --- | --- | --- | --- | --- | --- | --- | --- | --- | --- | --- | --- | --- | --- | --- | --- | --- | --- | --- | --- | --- | --- | --- | --- |
|  |  |  | Stacking | | | | | Watson Crick-Watson Crick | | | Hoogsteen-Hoogsteen | | | Sugar Edge-Sugar Edge | | | Watson Crick-Hoogsteen | | | Watson Crick-Sugar Edge | | | Hoogsteen-Sugar Edge | | |
|  |  |  | Upward | Downward | Inward | Outward | Total | Cis | Trans | Total | Cis | Trans | Total | Cis | Trans | Total | Cis | Trans | Total | Cis | Trans | Total | Cis | Trans | Total |
| MUMBO | 1CX0 | B | 0.896 | 1.000 | 0.000 | 0.655 | 0.870 | 0.909 | 0.000 | 0.909 | 0.000 | 0.000 | 0.000 | 0.000 | 1.000 | 1.000 | 0.000 | 0.000 | 0.000 | 0.000 | 0.000 | 0.000 | 0.000 | 0.000 | 0.000 |
|  | 1EXD | B | 0.857 | 0.000 | 0.408 | 0.603 | 0.791 | 0.873 | 0.000 | 0.853 | 0.000 | 0.000 | 0.000 | 0.000 | 0.000 | 0.000 | 0.000 | 0.707 | 0.707 | 0.000 | 0.000 | 0.000 | 0.000 | 0.816 | 0.577 |
|  | 1FFY | T | 0.882 | 0.000 | 0.408 | 0.764 | 0.836 | 0.957 | 0.000 | 0.938 | 0.000 | 1.000 | 1.000 | 0.000 | 0.000 | 0.000 | 0.000 | 0.816 | 0.816 | 0.000 | 1.000 | 1.000 | 0.000 | 0.000 | 0.000 |
|  | 1GID | A | 0.891 | 0.866 | 0.676 | 0.767 | 0.863 | 0.855 | 0.707 | 0.847 | 0.000 | 0.000 | 0.000 | 0.000 | 0.816 | 0.707 | 0.400 | 1.000 | 0.571 | 0.000 | 0.000 | 0.000 | 1.000 | 0.816 | 0.845 |
|  | 1I6U | C | 0.885 | 0.000 | 1.000 | 1.000 | 0.900 | 1.000 | 0.000 | 0.968 | 0.000 | 0.000 | 0.000 | 0.000 | 0.000 | 0.000 | 0.000 | 0.000 | 0.000 | 0.000 | 0.000 | 0.000 | 0.000 | 0.000 | 0.000 |
|  | 1MMS | C | 0.963 | 1.000 | 0.707 | 0.913 | 0.950 | 0.970 | 1.000 | 0.972 | 0.000 | 0.000 | 0.000 | 0.000 | 1.000 | 1.000 | 0.667 | 0.000 | 0.667 | 0.000 | 0.000 | 0.000 | 1.000 | 1.000 | 1.000 |
|  | 1U0B | A | 0.897 | 0.000 | 1.000 | 0.603 | 0.847 | 0.854 | 1.000 | 0.861 | 0.000 | 0.000 | 0.000 | 0.000 | 0.000 | 0.000 | 0.500 | 1.000 | 0.800 | 0.000 | 0.000 | 0.000 | 0.000 | 1.000 | 1.000 |
|  | 1UN6 | E | 0.911 | 0.000 | 1.000 | 0.894 | 0.913 | 0.973 | 0.000 | 0.973 | 0.000 | 1.000 | 1.000 | 0.000 | 0.000 | 0.000 | 0.000 | 0.000 | 0.000 | 0.000 | 0.000 | 0.000 | 1.000 | 0.000 | 0.707 |
|  | 1WZ2 | C | 0.750 | 1.000 | 0.000 | 0.535 | 0.707 | 0.750 | 0.000 | 0.735 | 0.000 | 0.000 | 0.000 | 0.000 | 0.000 | 0.000 | 0.000 | 0.816 | 0.577 | 0.000 | 0.000 | 0.000 | 0.000 | 0.000 | 0.000 |
|  | 1Y0Q | A | 0.796 | 0.000 | 0.516 | 0.564 | 0.744 | 0.774 | 0.000 | 0.768 | 0.000 | 0.000 | 0.000 | 0.000 | 0.577 | 0.408 | 0.000 | 0.354 | 0.169 | 0.408 | 0.000 | 0.333 | 0.000 | 0.600 | 0.600 |
|  | Overall | | 0.862 | 0.783 | 0.624 | 0.674 | 0.826 | 0.866 | 0.507 | 0.858 | 0.000 | 0.816 | 0.577 | 0.000 | 0.772 | 0.668 | 0.355 | 0.700 | 0.525 | 0.354 | 0.447 | 0.338 | 0.548 | 0.728 | 0.682 |
| RNAfitme | 1CX0 | B | 0.859 | 0.000 | 0.000 | 0.676 | 0.828 | 0.813 | 0.000 | 0.813 | 0.000 | 0.000 | 0.000 | 0.000 | 0.000 | 0.000 | 0.000 | 0.000 | 0.000 | 0.000 | 0.000 | 0.000 | 0.000 | 0.000 | 0.000 |
|  | 1EXD | B | 0.812 | 0.000 | 0.408 | 0.674 | 0.766 | 0.789 | 0.000 | 0.771 | 0.000 | 0.000 | 0.000 | 0.000 | 0.000 | 0.000 | 0.000 | 0.500 | 0.500 | 0.000 | 0.000 | 0.000 | 0.000 | 0.707 | 0.408 |
|  | 1FFY | T | 0.931 | 0.000 | 0.707 | 0.577 | 0.864 | 0.843 | 1.000 | 0.850 | 0.000 | 0.000 | 0.000 | 0.000 | 0.000 | 0.000 | 0.000 | 0.816 | 0.816 | 0.000 | 0.000 | 0.000 | 0.000 | 0.000 | 0.000 |
|  | 1GID | A | 0.888 | 0.000 | 0.632 | 0.460 | 0.817 | 0.866 | 0.000 | 0.858 | 0.000 | 0.000 | 0.000 | 0.000 | 0.577 | 0.500 | 0.671 | 0.707 | 0.676 | 0.000 | 0.000 | 0.000 | 0.000 | 0.471 | 0.436 |
|  | 1I6U | C | 0.902 | 0.000 | 0.000 | 0.408 | 0.843 | 0.933 | 0.000 | 0.933 | 0.000 | 0.000 | 0.000 | 0.000 | 0.000 | 0.000 | 0.000 | 0.000 | 0.000 | 0.000 | 0.000 | 0.000 | 0.000 | 0.000 | 0.000 |
|  | 1MMS | C | 0.900 | 0.000 | 0.707 | 0.408 | 0.821 | 0.843 | 0.000 | 0.819 | 0.000 | 0.000 | 0.000 | 0.000 | 0.000 | 0.000 | 0.816 | 0.000 | 0.816 | 0.000 | 0.000 | 0.000 | 0.000 | 1.000 | 0.816 |
|  | 1U0B | A | 0.909 | 0.000 | 0.707 | 0.853 | 0.891 | 0.827 | 0.707 | 0.813 | 0.000 | 0.000 | 0.000 | 0.000 | 0.000 | 0.000 | 0.000 | 0.000 | 0.000 | 0.000 | 0.000 | 0.000 | 0.000 | 1.000 | 1.000 |
|  | 1UN6 | E | 0.881 | 0.000 | 0.707 | 0.671 | 0.852 | 0.889 | 0.000 | 0.889 | 0.000 | 1.000 | 1.000 | 0.000 | 0.000 | 0.000 | 0.000 | 0.000 | 0.000 | 0.000 | 0.000 | 0.000 | 0.000 | 0.000 | 0.000 |
|  | 1WZ2 | C | 0.778 | 0.000 | 0.000 | 0.707 | 0.754 | 0.826 | 0.000 | 0.810 | 0.000 | 0.000 | 0.000 | 0.000 | 0.000 | 0.000 | 0.000 | 0.333 | 0.333 | 0.000 | 0.000 | 0.000 | 0.000 | 0.000 | 0.000 |
|  | 1Y0Q | A | 0.849 | 0.000 | 0.408 | 0.676 | 0.807 | 0.788 | 0.000 | 0.782 | 0.000 | 0.000 | 0.000 | 1.000 | 0.500 | 0.632 | 0.000 | 0.289 | 0.200 | 0.000 | 1.000 | 0.408 | 0.000 | 0.316 | 0.258 |
|  | Overall | | 0.868 | 0.000 | 0.552 | 0.630 | 0.820 | 0.833 | 0.436 | 0.826 | 0.000 | 0.577 | 0.500 | 0.500 | 0.333 | 0.375 | 0.533 | 0.397 | 0.449 | 0.000 | 0.447 | 0.169 | 0.000 | 0.553 | 0.431 |
| MMB | 1CX0 | B | 0.667 | 0.000 | 0.000 | 0.000 | 0.599 | 0.556 | 0.000 | 0.556 | 0.000 | 0.000 | 0.000 | 0.000 | 0.000 | 0.000 | 0.000 | 0.000 | 0.000 | 0.000 | 0.000 | 0.000 | 0.000 | 0.000 | 0.000 |
|  | 1EXD | B | 0.721 | 0.000 | 0.577 | 0.302 | 0.651 | 0.630 | 0.000 | 0.591 | 0.000 | 0.000 | 0.000 | 0.000 | 0.000 | 0.000 | 0.000 | 0.408 | 0.354 | 0.000 | 0.000 | 0.000 | 0.000 | 0.408 | 0.333 |
|  | 1FFY | T | 0.775 | 0.000 | 0.000 | 0.408 | 0.701 | 0.677 | 0.000 | 0.663 | 0.000 | 0.000 | 0.000 | 0.000 | 0.000 | 0.000 | 0.000 | 0.289 | 0.258 | 0.000 | 0.000 | 0.000 | 0.000 | 0.000 | 0.000 |
|  | 1GID | A | 0.73 | 0.000 | 0.000 | 0.566 | 0.675 | 0.742 | 0.000 | 0.735 | 0.000 | 0.000 | 0.000 | 0.000 | 0.577 | 0.500 | 0.000 | 0.000 | 0.000 | 0.000 | 0.000 | 0.000 | 0.000 | 0.577 | 0.535 |
|  | 1I6U | C | 0.734 | 0.000 | 0.000 | 0.577 | 0.685 | 0.683 | 0.000 | 0.683 | 0.000 | 0.000 | 0.000 | 0.000 | 0.000 | 0.000 | 0.000 | 0.000 | 0.000 | 0.000 | 0.000 | 0.000 | 0.000 | 0.000 | 0.000 |
|  | 1MMS | C | 0.749 | 0.000 | 0.000 | 0.289 | 0.672 | 0.542 | 0.000 | 0.527 | 0.000 | 0.000 | 0.000 | 0.000 | 0.000 | 0.000 | 0.577 | 0.000 | 0.577 | 0.000 | 0.000 | 0.000 | 0.000 | 0.707 | 0.577 |
|  | 1U0B | A | 0.823 | 0.000 | 0.000 | 0.674 | 0.761 | 0.798 | 1.000 | 0.808 | 0.000 | 0.000 | 0.000 | 0.000 | 0.000 | 0.000 | 0.000 | 0.000 | 0.000 | 0.000 | 0.000 | 0.000 | 0.000 | 1.000 | 1.000 |
|  | 1UN6 | E | 0.801 | 0.000 | 0.000 | 0.447 | 0.719 | 0.596 | 0.000 | 0.596 | 0.000 | 0.000 | 0.000 | 0.000 | 0.000 | 0.000 | 0.000 | 0.000 | 0.000 | 0.000 | 0.000 | 0.000 | 0.000 | 0.000 | 0.000 |
|  | 1WZ2 | C | 0.774 | 0.000 | 0.000 | 0.500 | 0.727 | 0.693 | 0.000 | 0.653 | 0.000 | 0.000 | 0.000 | 0.000 | 0.000 | 0.000 | 0.000 | 0.577 | 0.289 | 0.000 | 0.000 | 0.000 | 0.000 | 0.000 | 0.000 |
|  | 1Y0Q | A | 0.765 | 0.000 | 0.577 | 0.701 | 0.741 | 0.744 | 0.000 | 0.731 | 0.000 | 0.000 | 0.000 | 0.000 | 0.707 | 0.316 | 0.000 | 0.000 | 0.000 | 0.000 | 1.000 | 0.333 | 0.000 | 0.000 | 0.000 |
|  | Overall | | 0.754 | 0.000 | 0.195 | 0.533 | 0.700 | 0.691 | 0.189 | 0.679 | 0.000 | 0.000 | 0.000 | 0.000 | 0.408 | 0.250 | 0.075 | 0.208 | 0.141 | 0.000 | 0.158 | 0.109 | 0.000 | 0.361 | 0.308 |

# Table S3. Base pair-specific details of the INF values reported in Table 3 of the main text.

|  | **Number of individual structures/protein data bank entries^a^** | **Number of nucleotides** | **Number of pyrimidine nucleotides** | **Number of purine nucleotides** | **Number of dA or A nucleotides** | **Number of**  **dT or U nucleotides** | **Number of dG or G nucleotides** | **Number of dC or C nucleotides** |
| --- | --- | --- | --- | --- | --- | --- | --- | --- |
|  |  |  |  |  |  |  |  |  |
| **DNA structures** | 1,000 | 18,686 | 8,458 | 10,228 | 2,697 | 3,059 | 7,531 | 5,399 |
| **DNA-protein complexes** | 2,180 | 74,955 | 37,380 | 37,575 | 17,042 | 17,496 | 20,533 | 19,884 |
| **Grand total of deoxyribonucleotide-containing structures/deoxyribo-nucleotides** | **3,180** | **93,641** | **45,838** | **47,803** | **19,739** | **20,555** | **28,064** | **25,283** |
|  |  |  |  |  |  |  |  |  |
| **RNA structures** | 520 | 25,028 | 11,437 | 13,591 | 5,375 | 4,644 | 8,216 | 6,793 |
| **RNA-protein complexes** | 818 | 57,772 | 25,984 | 31,788 | 14,813 | 11,827 | 16,975 | 14,157 |
| **Grand total of ribo-nucleotide-containing structures/ribonucleotides** | **1,338** | **82,800** | **37,421** | **45,379** | **20,188** | **16,471** | **25,191** | **20,950** |

# Table S4. Dataset composition for the statistical analysis of pseudorotation angle P and torsion angle χ. ^a^Criteria for the inclusion of structures into the list: Structures solved by X-ray crystallography with resolutions better than 2.5 Å and released between January 2010 and January 2021.

|  | **Number of structures retrieved from protein data bank^a^** | **Number of curated structures** | **Number of atom coordinate spheres** | **Average number of spheres used for validation purposes** |
| --- | --- | --- | --- | --- |
| **Protein-DNA complexes** | 1589 | 686 | 3469 | ~600 |
| **Protein-RNA complexes** | 446 | 275 | 976 |  |

# Table S5. Reference dataset composition for the validation of the nucleoside rotamer library implementation and nucleotide selection process in MUMBO. ^a^Criteria for the inclusion of structures into the list: Structures solved by X-ray crystallography with resolutions better than 2.0 Å and released before February 2023 containing either DNA-protein or RNA-protein complexes. In order to save computational resources, only 600 of the 4445 spheres were used for validation purposes.

| **Nucleotide** | **Total number of nucleotides** | **Ribose conformation** | **Number of nucleotides** |
| --- | --- | --- | --- |
| dA | 19739 | 2'-endo-like | 17104 |
|  |  | 3'-endo-like | 2635 |
| dC | 25283 | 2'-endo-like | 19257 |
|  |  | 3'-endo-like | 6026 |
| dG | 28064 | 2'-endo-like | 22887 |
|  |  | 3'-endo-like | 5177 |
| dT | 20555 | 2'-endo-like | 17581 |
|  |  | 3'-endo-like | 2974 |
| A | 20188 | 2'-endo-like | 3712 |
|  |  | 3'-endo-like | 16476 |
| C | 20950 | 2'-endo-like | 1406 |
|  |  | 3'-endo-like | 19544 |
| G | 25191 | 2'-endo-like | 3005 |
|  |  | 3'-endo-like | 22186 |
| U | 16471 | 2'-endo-like | 3282 |
|  |  | 3'-endo-like | 13189 |

# Table S6. Dataset composition for GMM fitting.


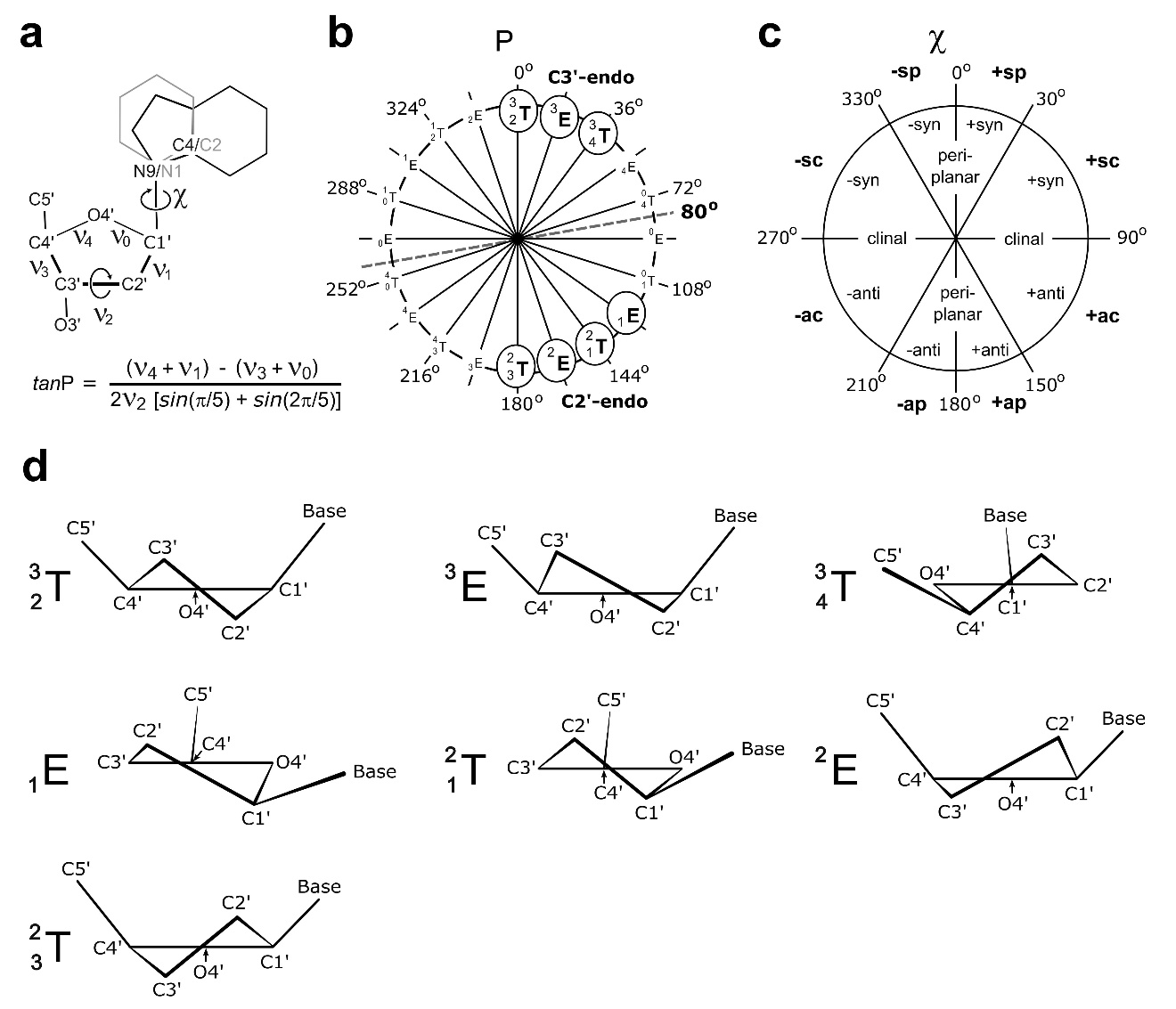


# Figure S1. Torsion angle χ and pseudorotation angle P. (a) Definition of the torsion angle χ and the pseudorotation angle P. The torsion angle χ describes the orientation of the nucleobase with respect to the ribose/deoxyribose ring. The torsion angle is defined *via* the atoms O4’-C1’-N9-C4 for purine and O4’-C1’-N1-C2 for pyrimidine nucleobases. The pseudorotation angle P is calculated from the five torsion angles (ν0,ν1,ν2,ν3,ν4,ν5) present in the five-membered furanose ring or the ribose/deoxyribose sugar moiety. Together with the pseudorotation amplitude τ_m_, P allows for an unambiguous characterization of the puckering conformation of the furanose ring [1]. (b) Pseudorotation angle P and sugar ring puckering. T stands for the twist and E for the envelope conformation. ^3^_2_T stands for C3’-endo-C2’-exo twist conformation and corresponds to a pseudorotation angle of 0°. ^2^E describes the C2’-endo conformation commonly observed in B-DNA. Highlighted by circles are those conformations that are included in program MUMBO during nucleotide building. (c) Classification of the χ torsion angle [2]. The classification used throughout the manuscript is highlighted in bold. (d) Illustration of the ring puckering conformations highlighted with circles in panel (b).


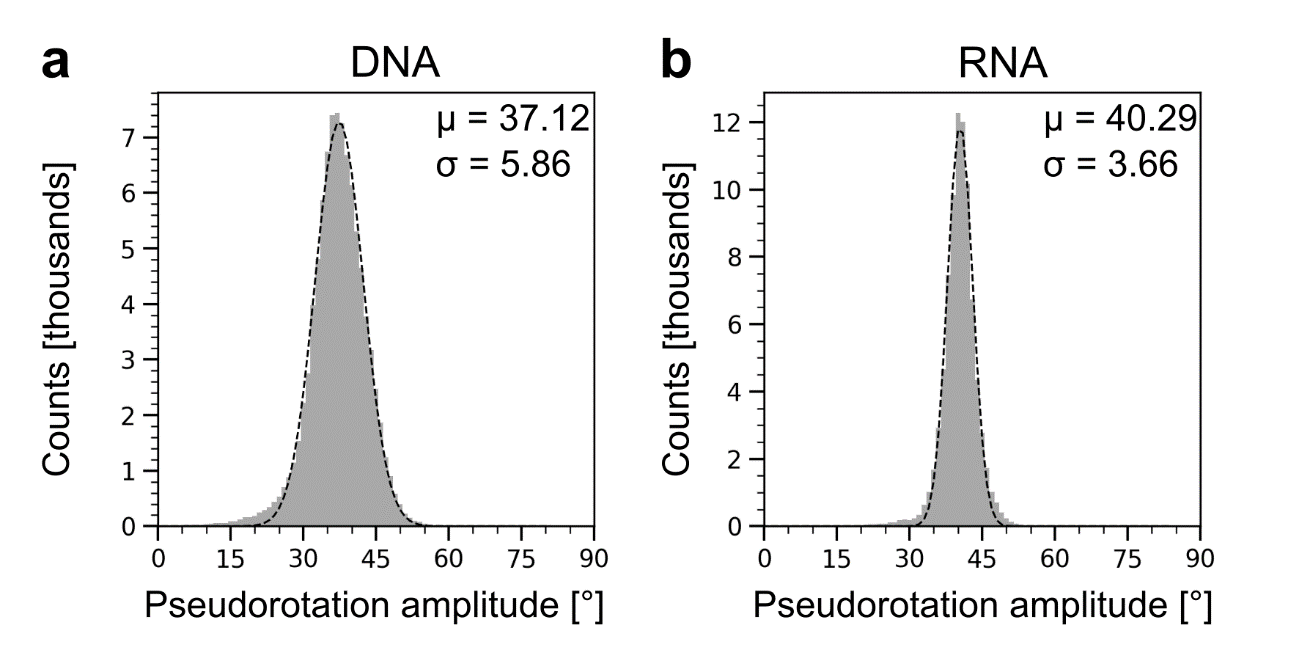


# Figure S2. Pseudorotation amplitude τ_M_ distributions. (a) in deoxyribonucleotides and (b) ribonucleotides. Gaussian curves fitted for estimation of means and standard deviations are shown in dashed line.


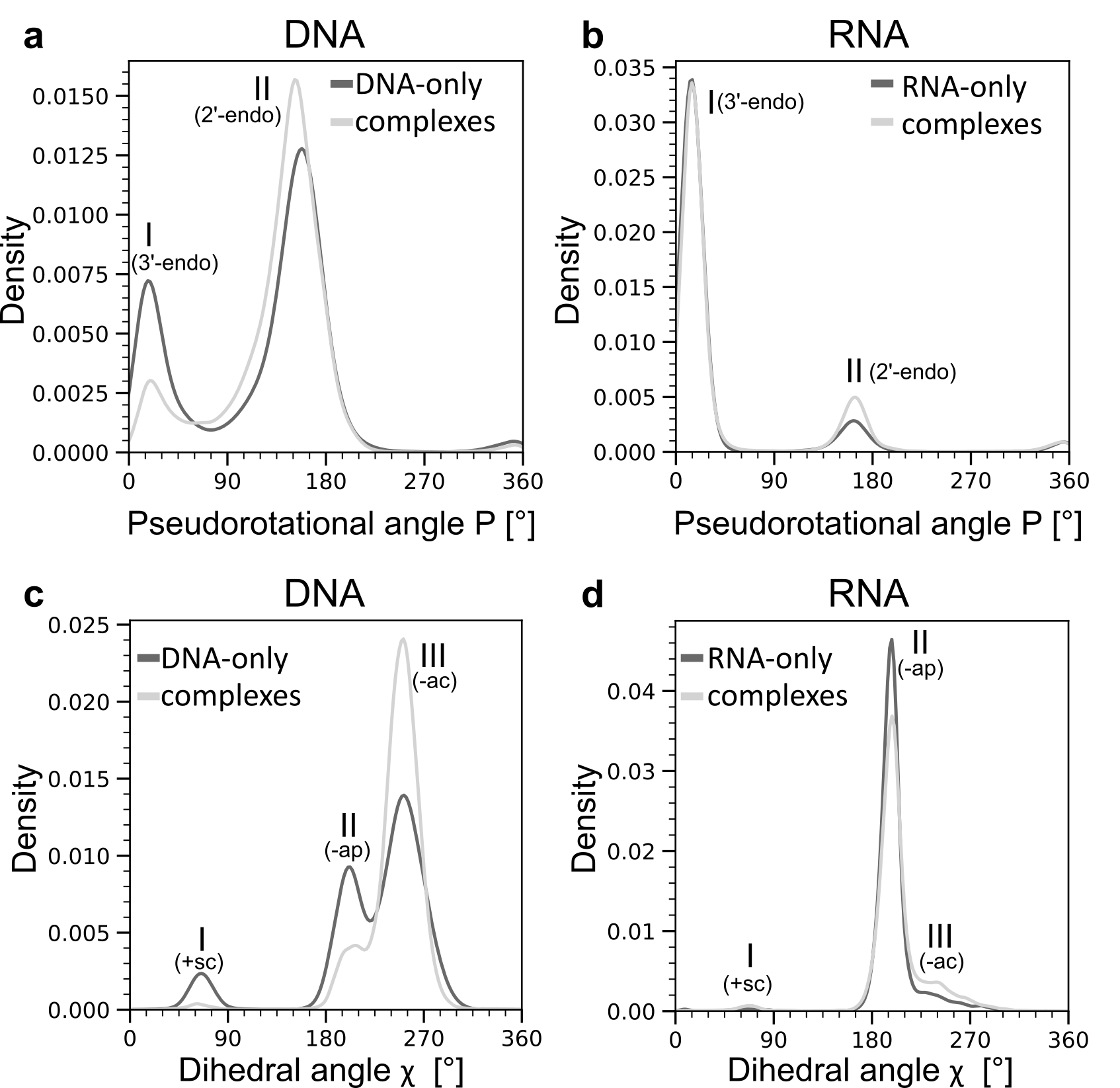


# Figure S3. Differences in pseudorotation angle P and dihedral angle χ distributions in nucleotides from structures of individual polynucleotides and polynucleotide-protein complexes: (a) P angles in deoxyribonucleotides, (b) P angles in ribonucleotides, (c) dihedral angles χ in deoxyribonucleotides and (d) angles χ in ribonucleotides.


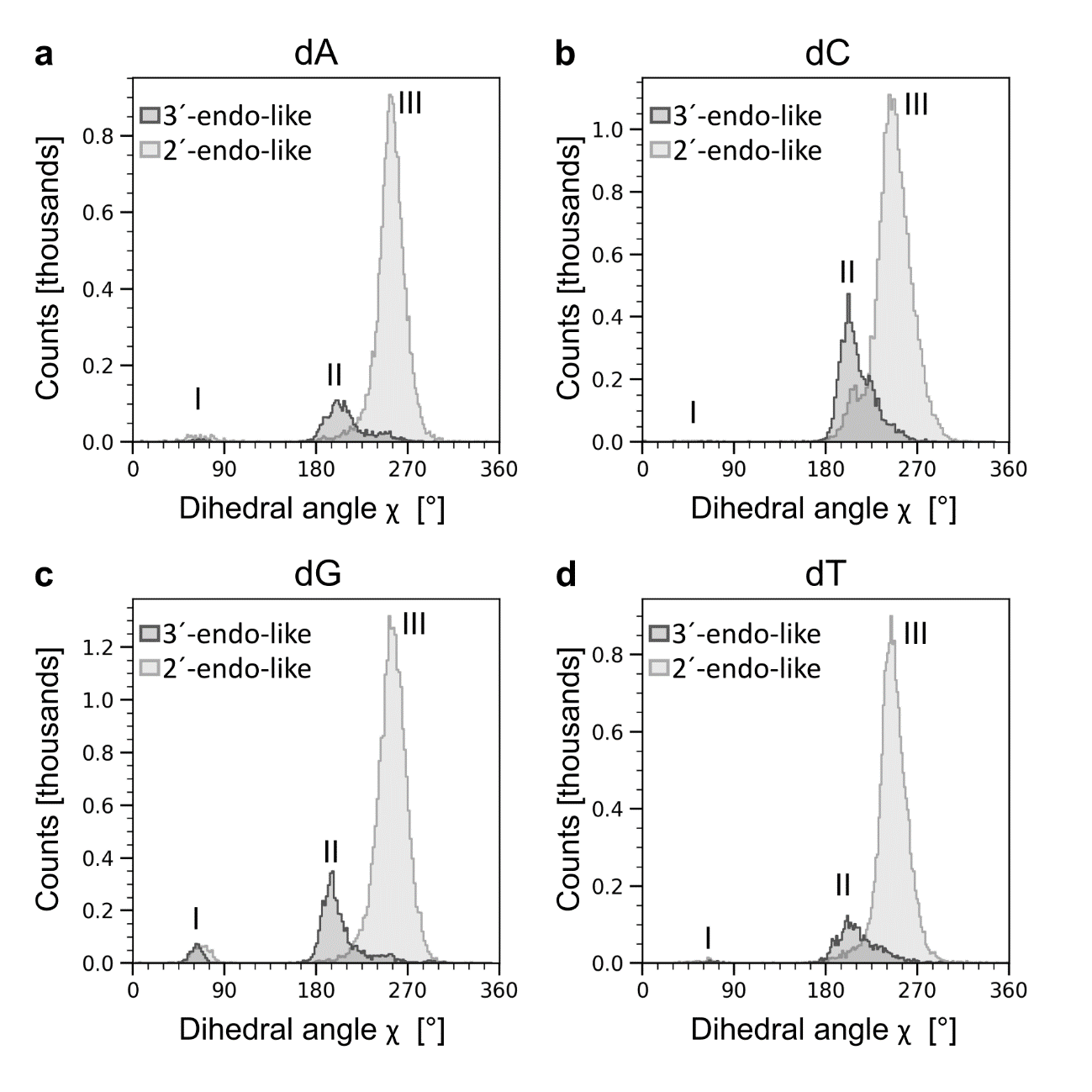


# Figure S4. P angle-dependent distributions of χ angles in DNA-containing structures: (a) χ angle distributions in deoxyadenosine, (b) χ angle distributions in deoxycytidine, (c) χ angle distributions in deoxyguanosine and (d) χ angle distributions in deoxythymidine.


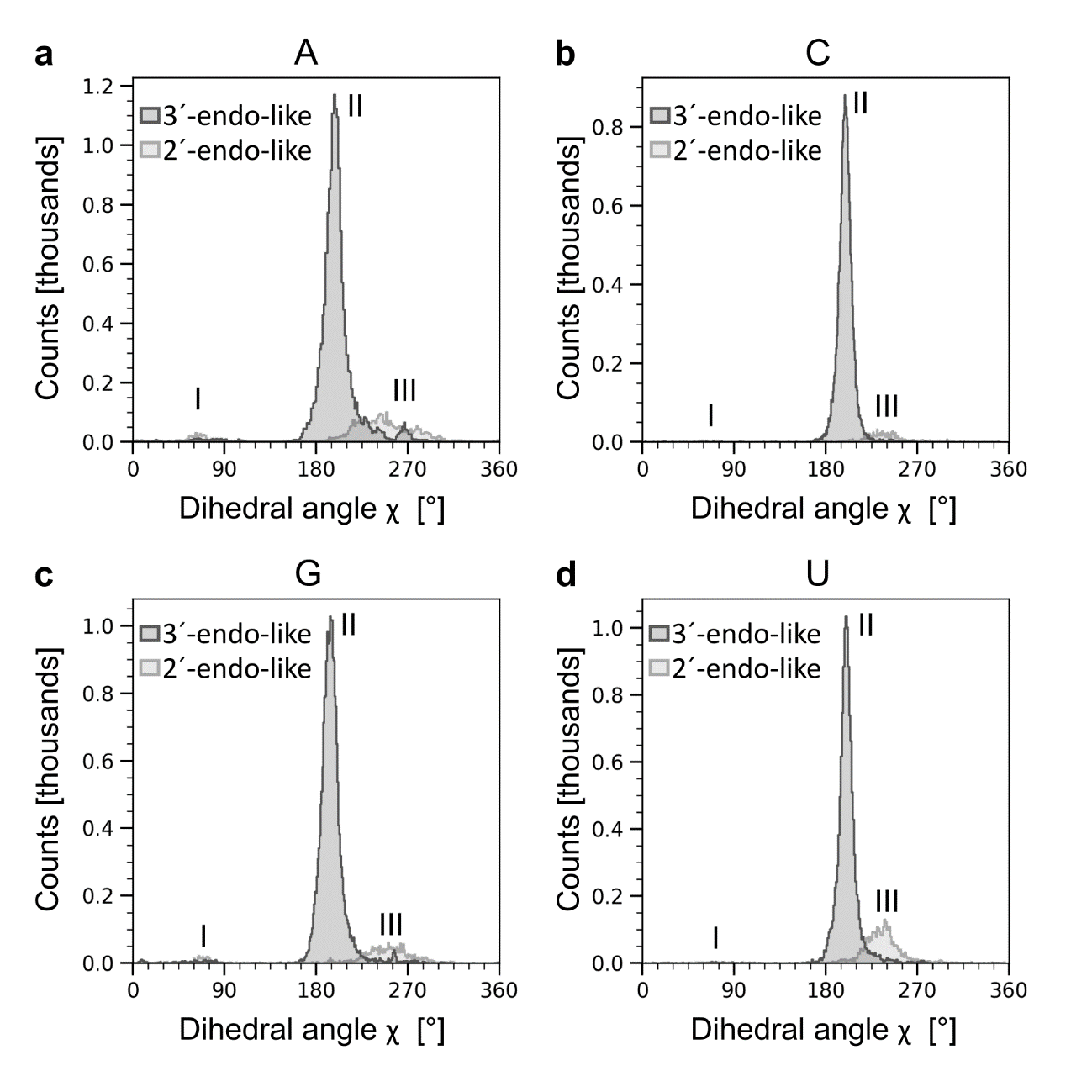


# Figure S5. P angle-dependent χ angle distributions in RNA-containing structures: (a) χ angle distributions in adenosine, (b) χ angle distributions in cytidine, (c) χ angle distributions in guanosine and (d) χ angle distributions in uridine.

**
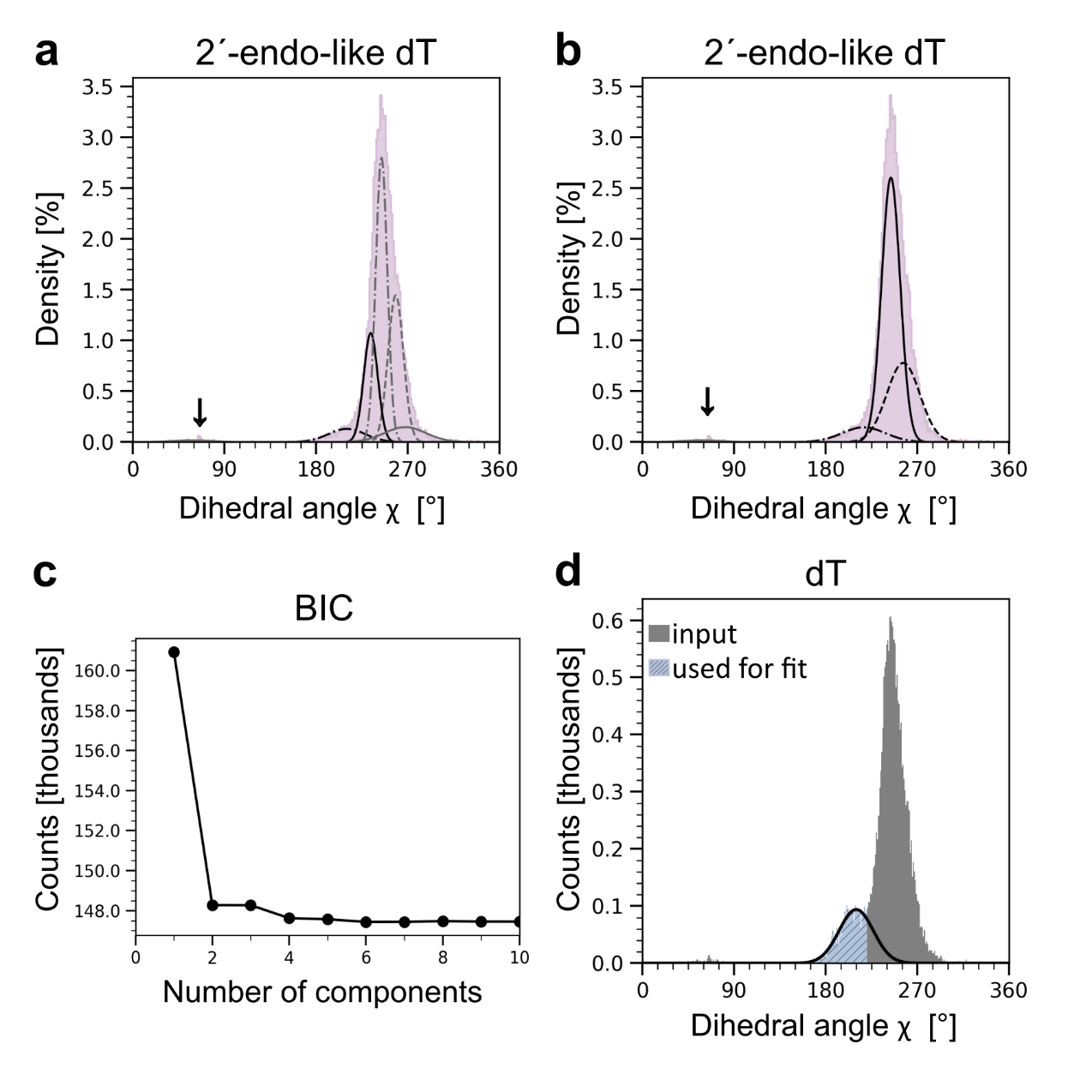
**

# Figure S6. Illustration of the methods used to derive preferred nucleobase orientations, i.e. rotamer libraries. (a) Gaussian mixture model with 6 components (as suggested by the overall minimum BIC value estimation, see panel C) fitted to the χ angle distribution for 2´-endo-like dT. Arrow points at the curve fitted to the +syn peak. (b) Gaussian mixture model with 4 components (as suggested by the first local minimum BIC value estimation with additional selection criteria) fitted to the χ angle distribution for 2´-endo-like dT. Arrow points at the curve fitted to the +syn peak. (c) Graphical representation of the BIC values of Gaussian mixture models with different numbers of components fitted to the P-dependent χ angle distribution for 2´-endo-like dT. (d) In order to obtain a simpler library with fewer rotamers (basic library), individual peaks are modelled by single Gaussian curves, as illustrated here for the P-independent χ angle distribution of dT.


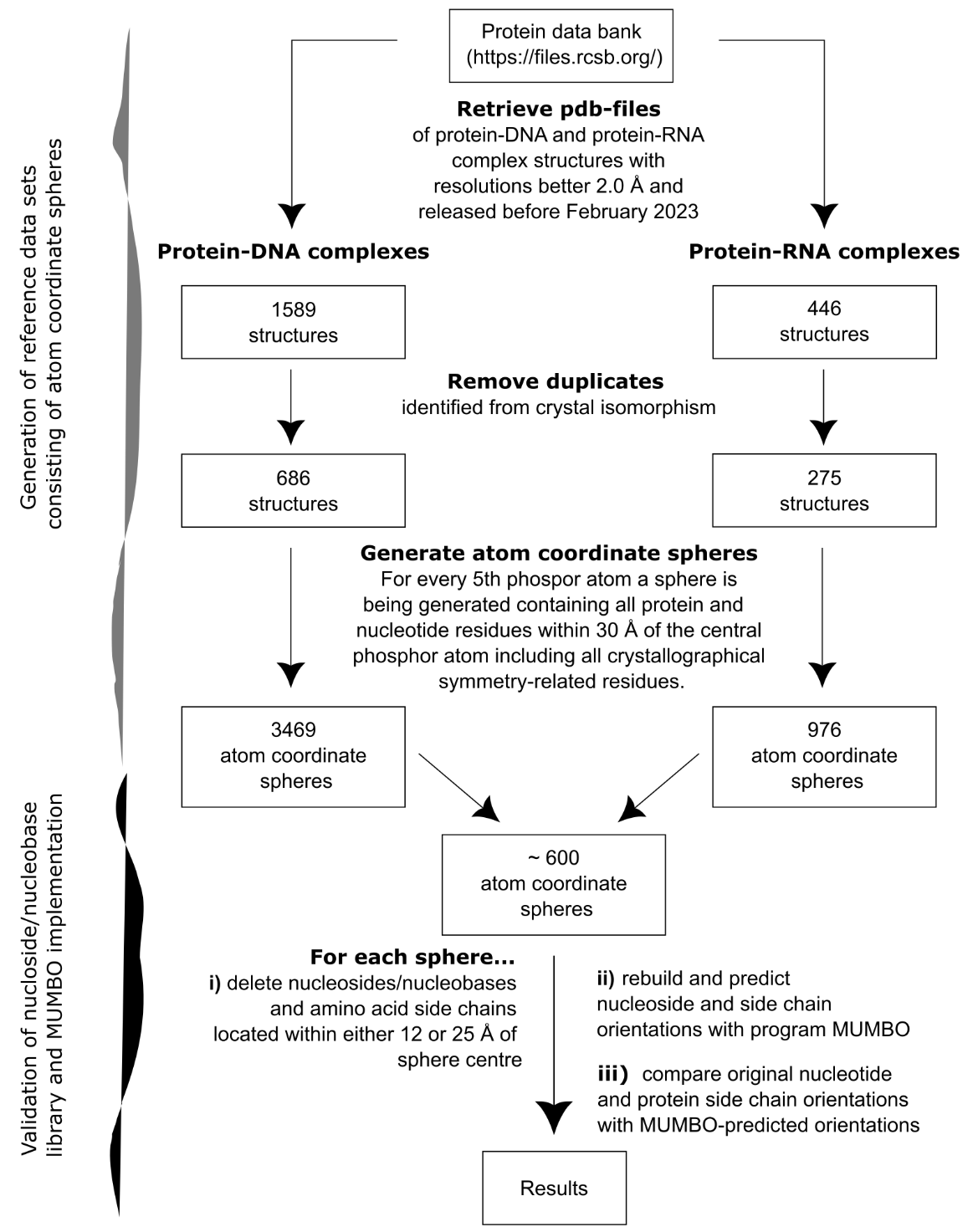


# Figure S7. Flow scheme for the generation of the DNA/RNA reference dataset used for the validation of the nucleoside rotamer library implementation and nucleotide selection process in MUMBO. Please see also Table S3 and Figure S8.


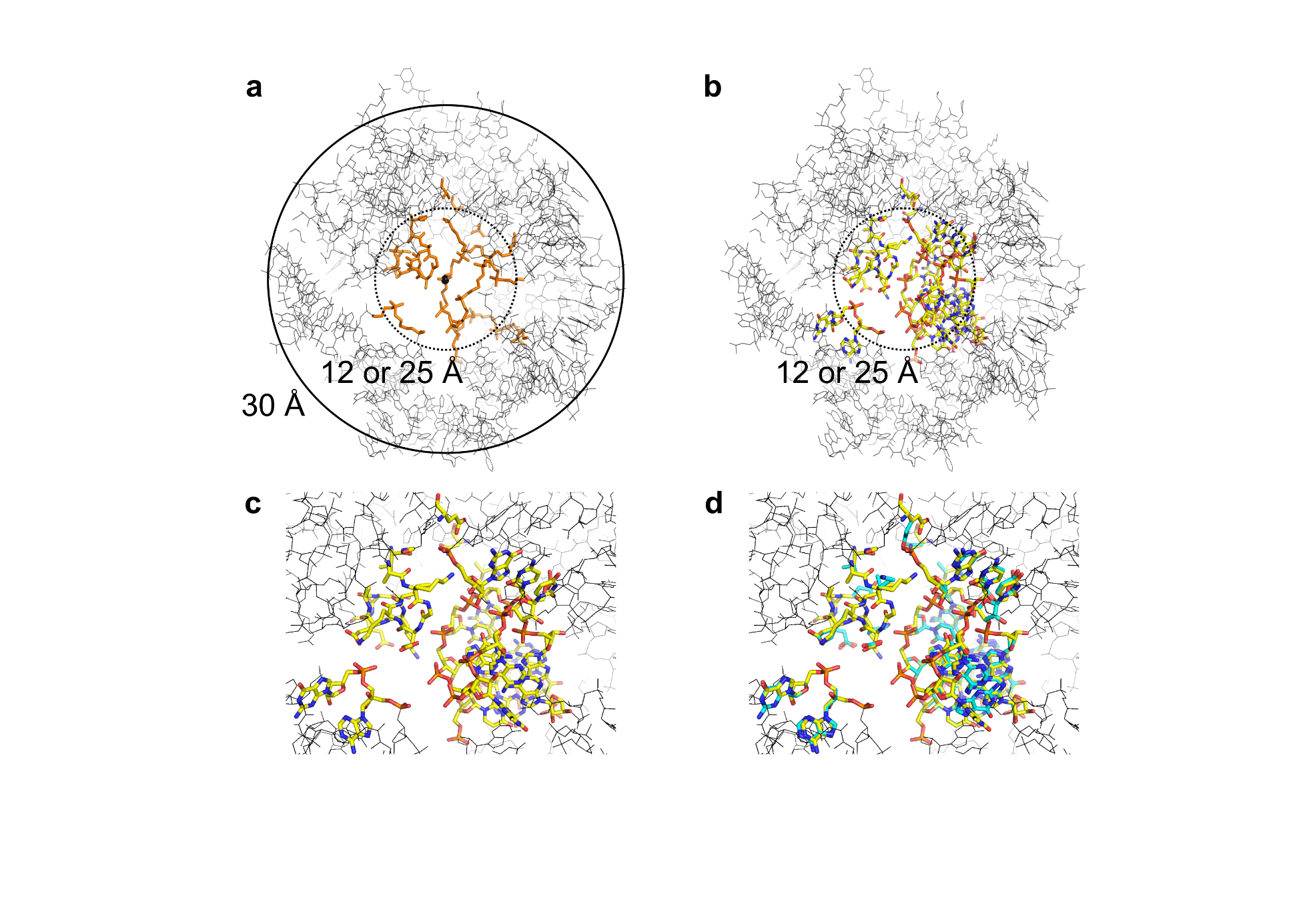


# Figure S8. Validation of the nucleoside rotamer libraries and their implementation into program MUMBO. (a) For validation purposes, amino acids side-chains and nucleotides are being rebuild if located within 12 or 25 Å of the centre of 30 Å wide reference coordinate spheres. (b) MUMBO-generated amino acid side-chain and nucleotide packing using the original protein and polynucleotide sequence as input (in yellow). (c) Close-up view of panel (B). (d) Comparison of the MUMBO-generated packing (in yellow) with the original packing (in blue) as present in the reference dataset.

# Supplementary references

1 Altona, C. & Sundaralingam, M. Conformational analysis of the sugar ring in nucleosides and nucleotides. A new description using the concept of pseudorotation. *J Am Chem Soc* **94**, 8205-8212, doi:10.1021/ja00778a043 (1972).

2 Saenger, W. *Principles of nucleic acid structure*. (Springer-Verlag, 1984).
